# A Hybrid Nonlinear Whale Optimization Algorithm with Sine Cosine for Global Optimization

**DOI:** 10.3390/biomimetics9100602

**Published:** 2024-10-07

**Authors:** Yubao Xu, Jinzhong Zhang

**Affiliations:** School of Electrical and Photoelectronic Engineering, West Anhui University, Lu’an 237012, China

**Keywords:** whale optimization algorithm, sine cosine algorithm, nonlinear, benchmark functions, engineering designs

## Abstract

The whale optimization algorithm (WOA) is constructed on a whale’s bubble-net scavenging pattern and emulates encompassing prey, bubble-net devouring prey, and stochastic capturing for prey to establish the global optimal values. Nevertheless, the WOA has multiple deficiencies, such as restricted precision, sluggish convergence acceleration, insufficient population variety, easy premature convergence, and restricted operational efficiency. The sine cosine algorithm (SCA) constructed on the oscillation attributes of the cosine and sine coefficients in mathematics is a stochastic optimization methodology. The SCA upgrades population variety, amplifies the search region, and accelerates international investigation and regional extraction. Therefore, a hybrid nonlinear WOA with SCA (SCWOA) is emphasized to estimate benchmark functions and engineering designs, and the ultimate intention is to investigate reasonable solutions. Compared with other algorithms, such as BA, CapSA, MFO, MVO, SAO, MDWA, and WOA, SCWOA exemplifies a superior convergence effectiveness and greater computation profitability. The experimental results emphasize that the SCWOA not only integrates investigation and extraction to avoid premature convergence and realize the most appropriate solution but also exhibits superiority and practicability to locate greater computation precision and faster convergence speed.

## 1. Introduction

Systematic optimization executes distinct logical reasoning strategies to eliminate the high-dimensional sophisticated mathematical difficulties and derive international optimal solutions. The ultimate intention is to attain the most incredible quality, efficiency, risk, and profit. Conventional numerical methodologies exhibit deficiencies in low computational productivity, sluggish convergence acceleration, easy premature convergence, lengthy activation consumption, and substantial combinatorial explosion. Nevertheless, the attributes of the swarm intelligence algorithms comprise high computation precision, great flexibility and adaptability, extensive population variety, excellent self-management, and convenient algorithmic combination, such as the bat algorithm (BA) [1], capuchin search algorithm (CapSA) [2], moth flame optimization (MFO) [3], multi-verse optimization (MVO) [4], smell agent optimization (SAO) [5], and movable damped wave algorithm (MDWA) [6].

Uzer et al. executed a modified hybrid WOA with particle swarm optimization to navigate the mathematical equations, and the methodology received a reliable international exploratory approach to discover the most advantageous optimal solutions [7]. Elmogy et al. introduced an adaptive nonlinear WOA to navigate the mathematical equations, and the methodology featured attractive sustainability and authenticity to promote convergence acceleration and elevate computational effectiveness [8]. Yang et al. integrated WOA and grey wolf optimization to navigate the mathematical equations, and the methodology exhibited extraordinary superiority and durability to maximize convergence velocity and computational productivity [9]. Zhang et al. constructed a modified WOA to navigate the proportion integration differentiation, and the methodology utilized differential filtering to accelerate the whale’s devouring proficiency and estimate desirable optimal solutions [10]. Quan et al. invented a modified WOA to simplify the thermoelectric generator, and the methodology maintained essential superiority and practicability to accomplish more substantial conversion accuracy and computational productivity [11]. Wei et al. introduced a neighborhood search WOA to navigate the mathematical equations, and the methodology exhibited exceptional reliability and stability to locate the most advantageous optimal solutions [12]. Wu et al. constructed a WOA to recognize microgrid faults, and the methodology confirmed substantial identification effectiveness and computation proficiency [13]. Fan et al. devised a WOA with a fuzzy-weighted framework to estimate body fat, and the methodology exhibited outstanding consistency and superiority in both ascertaining the diagnosis and recognizing a suitable calculated remedy [14]. Nadimi-Shahraki et al. summarized the WOA’s mathematical structures, refinements, and hybridizations, and the methodology was executed systematically and comprehensively to identify frontier situations [15]. Mohite et al. established a hybrid WOA with rain optimization to navigate the resource allocation, and the methodology retained remarkable superiority and adaptability to inhibit premature convergence and maximize the intended resource [16]. Routa et al. designed a WOA to navigate the mathematical equations and anticipate the seismic reaction, and the methodology exhibited computational capability and attractive integration precision [17]. Chakraborty et al. implemented a modified WOA based on horizontal crossover and a cooperative scavenging procedure to navigate the feature selection, and the methodology displayed outstanding equilibrium and predictability to yield higher quality measures [18]. Kumar et al. delivered a WOA to navigate the quality service resource distribution, and the methodology depicted fantastic dependability and superiority in diminishing distinctive scheduling costs [19]. Zhang et al. integrated adaptive WOA with differential evolution to navigate mutant motion tracking, and the methodology maintained tremendous tracking competitiveness and acceptable computational quality [20]. Deng et al. explained a multi-strategy WOA to navigate the mathematical equations, and the methodology exhibited exceptional durability and trustworthy in regulating the desirable optimal solutions [21]. Li et al. deployed a hybrid WOA with a symbiotic organisms search algorithm to navigate the mathematical equations, and the methodology featured tremendous dependability and security to elevate computational productivity and upgrade integration precision [22]. Zhang et al. described a complex-valued encoding WOA to navigate the mathematical equations, and the methodology integrated international investigation and regional extraction to deliver more accurate computed solutions [23]. Zhang et al. invented a WOA to alleviate the weapon–target requirement, and the methodology exhibited exceptional battle precision and decision-making productivity [24]. Liu et al. integrated WOA with a reinforced investigation to navigate the mathematical equations, and the methodology conveyed influential investigation validity and completion effectiveness [25]. Lin et al. mentioned a WOA with a niching mechanism to navigate the mathematical equations, and the methodology displayed outstanding reliability and superiority in identifying the greatest optimal solutions [26]. To summarize, research on the WOA mainly focuses on the algorithm enhancement and algorithm application. For algorithm enhancement, the enhanced WOA utilizes a unique detection strategy and an efficient encoding mechanism or hybrid search algorithm to achieve complementary advantages and improve the overall solution efficiency, and is utilized to resolve the function optimization and engineering design. The enhanced WOA not only balances exploration and exploitation to avoid premature convergence but also exhibits strong stability and feasibility to accelerate the convergence speed and improve the calculation accuracy. For algorithm application, the enhanced WOA exhibits strong stability, robustness, feasibility, adaptability, stabilization, scalability, self-regulation, and parallelism to solve various large-scale and complex optimization problems, such as artificial intelligence, systems control, pattern recognition, resource allocation, engineering technology, and network communication, finance, and other fields. The modified GJO method exhibits strong adaptability and robustness to promote calculation accuracy and achieve the optimal solution.

The WOA, constructed on a whale’s bubble-net scavenging pattern, emulates encompassing prey, bubble-net devouring prey, and stochastic capturing for prey to explore international optimal solutions [27]. To remedy the inadequacies of the WOA’s restricted computational precision, sluggish convergence acceleration, easy premature convergence, and restricted operational efficiency, a hybrid nonlinear SCWOA has been implemented to navigate benchmark functions and engineering designs. The SCWOA integrates the SCA’s mathematical oscillation attributes with the WOA’s bubble-net scavenging pattern to elevate population variety and enlarge the investigation region. The experimental results emphasize that the SCWOA not only regulates international investigation and regional extraction to avert premature convergence and upgrade the convergence acceleration but also exhibits superiority and practicability to cultivate the most accurate optimal solutions and maximize computational productivity.

The article is segregated into the subsequent sections. Section 2 exhibits the WOA. Section 3 summarizes the SCWOA. The simulation assessment and outcome interpretation are clarified in Section 4. Conclusions and further investigation are outlined in Section 5.

## 2. WOA

**Figure 1:**
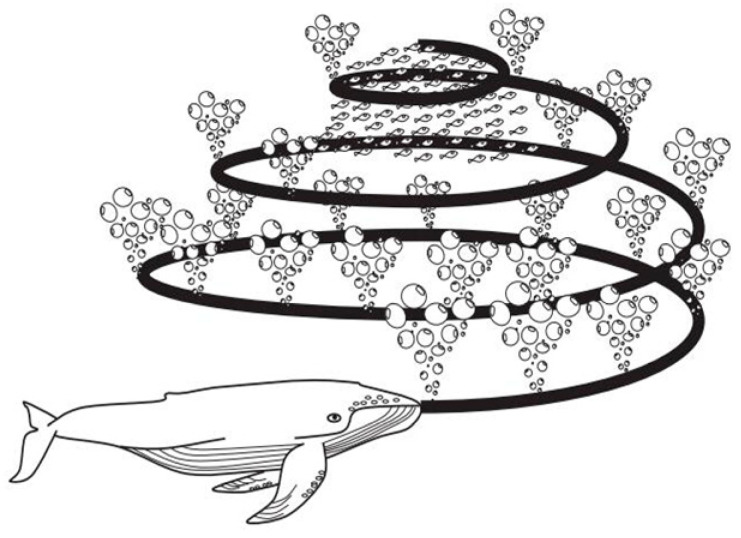
Bubble-net devouring motion.

The WOA exhibits the distinctive bubble-net scavenging pattern to summarize the mathematical framework, which comprises three anticipation strategies: encompassing prey, bubble-net devouring prey, and stochastic capturing for prey. Figure 1 portrays the bubble-net devouring motion.

### 2.1. Encompassing Prey

The latest fantastic whale is speculated to be the intended prey, and the leader whale integrates echolocation to convey documentation to other individuals, attacking the remaining whales to maneuver and encompass the prey. The structure is manufactured as follows:

(1) $$D=\left|C\cdot X^{*}(t)-X(t)\right|$$

(2) $$X(t+1)=X^{*}(t)-A\cdot D$$

where t symbolizes the most recent iteration, $X^{*}$ symbolizes the most appropriate location vector, X symbolizes the most recent location vector, || symbolizes the absolute quantity, and ⋅ symbolizes multiplying components. The A and C are manufactured as follows:

(3) A=2⋅a⋅r−a

(4) C=2⋅r

(5) a=2−2⋅t/T

where r symbolizes a disordered solution in $\left[0,1\right]$, a gradually declines from 2 to 0, and T symbolizes the highest iteration.

### 2.2. Bubble-Net Devouring Prey

The whale exploits the bubble-net devouring prey, which comprises two categories: swaying contraction encompassing prey and logarithmic spiral swallowing prey. Equations (3) and (5) accomplish the swaying contraction encompassing prey. The logarithmic spiral swallowing prey estimates the approximate separation between the intended prey and the most recent whale, and the whales spew uneven bubbles and traverse in a spiral motion to acquire the prey. The structure is manufactured as follows:

(6) $$D^{\prime }=\left|X^{*}(t)-X(t)\right|$$

(7) $$X(t+1)=D^{\prime }\cdot e^{bl}\cdot \cos(2\pi l)+X^{*}(t)$$

where $D^{\prime }$ symbolizes the route between the most recent whale and the most excellent prey, l symbolizes a disordered solution in $\left[-1,1\right]$, and b symbolizes an integer constant.

There is a 50% likelihood of identifying between swaying contraction encompassing prey and logarithmic spiral swallowing prey. The structure is manufactured as follows:

(8) $$X(t+1)=\left\{\begin{array}{l} X^{*}(t)-A\cdot D\;\;\;\;\;\;\;\;\;\;\;\;\;\;\;\;\;\;\;\;\;\;\;\;\;\;\;\;\;\;\;\;\;\;\;\;if\;\;\;\;p<0.5\\ D^{\prime }\cdot e^{bl}\cdot \cos(2\pi l)+X^{*}(t)\;\;\;\;\;\;\;\;\;\;\;\;\;\;\;\;if\;\;\;p\geq 0.5 \end{array}\right.$$

where p symbolizes a disordered solution in $\left[0,1\right]$.

### 2.3. Stochastic Capturing for Prey

If $\left|A\right|\geq 1$, the whale investigates an extensive search territory beyond the narrowing enclosure, one whale is automatically nominated as a reference individual, and the remaining whales will accumulate around this exact location. The structure is manufactured as follows:

(9) $$D=\left|C\cdot X_{rand}(t)-X(t)\right|$$

(10) $$X(t+1)=X_{rand}(t)-A\cdot D$$

where $X_{rand}$ symbolizes a disordered location vector.

Algorithm 1 comprises the WOA’s pseudocode version.
**Algorithm 1** WOA**Step 1.** Initialize population $X_{i}(i=1,2,\ldots ,n)$ **Step 2.** Investigate each attainable alternative’s fitness    $\mathrm{Diagnose}\;\mathrm{the}\;\mathrm{greatest}\;\mathrm{location}\;\mathrm{vector}\;X^{*}$ **Step 3.** while (t<T) do       **for** each attainable alternative       Customize a, A, C, l and p       **if1** (p<0.5)         $\;\mathrm{if}2\;(\left|A\right|<1)$
           Customize the attainable alternative’s location via Equation (2)         $\;\mathrm{else}\;\mathrm{if}2\;(\left|A\right|\geq 1)$          $\;\mathrm{Locate}\;a\;\mathrm{disordered}\;\mathrm{location}\;\mathrm{vector}\;X_{rand}$
           Customize the attainable alternative’s location via Equation (10)          **end if2**       **else if1** (p≥0.5)
           Customize the attainable alternative’s location via Equation (7)           **end if1**       **end for**
       Validate if any attainable alternative exists outside the search zone and readjust location       Investigate each attainable alternative’s fitness       $\mathrm{Customize}\;X^{*}$ if a superior location vector exists       t=t+1 **end while** $\mathrm{Retrieve}\;X^{*}$


## 3. SCWOA

The SCA exhibits multiple attributes of intuitive structure, fantastic robustness, enormous parallelism, convenient implementation, and substantial operational effectiveness. The synergistic benefits of investigation and extraction are accomplished by the productive combination of nonlinear WOA and SCA, which exhibits fantastic durability and adaptability to customize the integration precision.

### 3.1. Nonlinear WOA

The nonlinear control parameter modulates investigation and extraction to discover the most advantageous optimal solutions [28]. The structure is manufactured as

(11) $$W=2\cdot e^{-{\left(\frac{8t}{T}\right)}^{2}}$$

(12) $$X(t+1)=W\cdot X^{*}(t)-A\cdot D$$

(13) $$X(t+1)=D^{\prime }\cdot e^{bl}\cdot \cos(2\pi l)+W\cdot X^{*}(t)$$

(14) $$X(t+1)=W\cdot X_{rand}(t)-A\cdot D$$

### 3.2. SCA

The SCA utilizes the mathematical oscillation attributes of the cosine and sine coefficients to deliver more accurate computed solutions [29]. The waveform’s outward expansion symbolizes the international investigation and the fluctuation approaching the desired optimal solutions. The structure is manufactured as follows:

(15) $$X_{i}^{t+1}=\left\{\begin{array}{c} X_{i}^{t}+r_{1}\cdot \sin(r_{2})\cdot \left|r_{3}\cdot P_{i}^{t}-X_{i}^{t}\right|,\;\;\;\;\;\;r_{4}<0.5\\ X_{i}^{t}+r_{1}\cdot \cos(r_{2})\cdot \left|r_{3}\cdot P_{i}^{t}-X_{i}^{t}\right|,\;\;\;\;\;\;r_{4}\geq 0.5 \end{array}\right.$$

where $X_{i}^{t}$ symbolizes the most recent location, $X_{i}^{t+1}$ symbolizes the altered location, $P_{i}^{t}$ symbolizes the most appropriate location, $r_{1}$, $r_{2}$, $r_{3}$, $r_{4}$ symbolizes the disordered solutions, $r_{2}\in [0,2\pi ]$, $r_{3}\in [-2,2]$, $r_{4}\in [0,1]$, and || symbolizes the absolute quantity.

The amplitude conversion coefficient $r_{1}$ is manufactured as follows:

(16) $$r_{1}=a-t\cdot \frac{a}{T}$$

where a symbolizes a constant.

### 3.3. SCWOA

The SCWOA integrates the SCA’s mathematical oscillation attributes with the WOA’s bubble-net scavenging pattern to elevate convergence acceleration, augment population variety, and enlarge the investigation region. The SCWOA integrates investigation and extraction to maximize devouring proficiency and realize the most advantageous optimal solutions. It also exhibits superiority and practicability to furnish extraordinary battle precision and decision-making productivity. The SCWOA progressively contracts and hovers close to the desired solution by retaining the most recent fantastic leader whale’s location. The structure is manufactured as follows:

(17) $$X(t+1)=\left\{\begin{array}{c} X(t)+r_{1}\cdot \sin(r_{2})\cdot \left|r_{3}\cdot X^{*}(t)-X(t)\right|,\;\;\;\;\;\;r_{4}<0.5\\ X(t)+r_{1}\cdot \cos(r_{2})\cdot \left|r_{3}\cdot X^{*}(t)-X(t)\right|,\;\;\;\;\;\;r_{4}\geq 0.5 \end{array}\right.$$

where X symbolizes the most recent location vector, $r_{2}\in [0,2\pi ]$, $r_{3}\in [-2,2]$, $r_{4}\in [0,1]$, and $r_{1}$ diminishes continuously from 2 to 0.

Algorithm 2 comprises the SCWOA’s pseudocode version.
**Algorithm 2** SCWOA**Step 1.** Initialize population $X_{i}(i=1,2,\ldots ,n)$ **Step 2.** Investigate each attainable alternative’s fitness     $\mathrm{Diagnose}\;\mathrm{the}\;\mathrm{greatest}\;\mathrm{location}\;\mathrm{vector}\;X^{*}$ **Step 3. while** (t<T) do        **for** each attainable alternative        Customize a, A, C, l and p        **if1** (p<0.5)         $\mathrm{if}2\;(\left|A\right|<1)$
         The nonlinear strategy is introduced into the encompassing prey          Combine SCA with the encompassing prey          Customize the attainable alternative’s location via Equations (12) and (17)         $\mathrm{else}\;\mathrm{if}2\;(\left|A\right|\geq 1)$
         The nonlinear strategy is introduced into the stochastic capturing for prey         Combine SCA with the stochastic capturing for prey (exploration phase)         $\mathrm{Locate}\;a\;\mathrm{disordered}\;\mathrm{location}\;\mathrm{vector}\;X_{rand}$
         Customize the attainable alternative’s location via Equations (14) and (17)          **end if2**        **else if1** (p≥0.5)
         The nonlinear strategy is introduced into the bubble-net devouring prey         Combine SCA with the bubble-net devouring prey (exploitation phase)          Customize the attainable alternative’s location via Equations (13) and (17)         **end if1**       **end for**
       Validate if any attainable alternative exists outside the search zone and readjust the location       Investigate each attainable alternative’s fitness       $\mathrm{Customize}\;X^{*}$ if a superior location vector exists        t=t+1 **end while** $\mathrm{Retrieve}\;X^{*}$


### 3.4. Complexity Analysis

The computational complexity reveals the magnitude of reaction time advances with the input size. The big-O notation is a trustworthy and consistent procedure for substantially assessing the algorithm’s attributes and sophistication. Time complexity is a mathematical equation that yields an exhaustive evaluation of the computational expenditure. The SCWOA exhibits five phases: initialization, swaying and encompassing prey, bubble-net devouring prey, amending the attainable alternative’s location, and suspending adjudication. In SCWOA, the population magnitude is N, the highest iteration is T, and the question dimension is D. For initialization, it symbolizes O(N⋅D). For swaying and encompassing prey, bubble-net devouring prey, and amending the attainable alternative’s location, it symbolizes O(N⋅D⋅T). For suspending adjudication, it symbolizes O(1). Consequently, the ultimate time complexity of the SCWOA symbolizes O(N⋅D⋅T). Space complexity quantifies the amount of temporary storage space, and the maximum space complexity of the SCWOA symbolizes O(N⋅D). The SCWOA exhibits practicality and superiority in navigating the various multifaceted challenges.

## 4. Simulation Assessment and Results Interpretation

### 4.1. Experimental Configuration

Computational configuration is installed on a Windows 10 with an Intel Core i7-8750H 2.2 GHz CPU, a GTX1060, and 8 GB RAM.

### 4.2. Benchmark Functions

The three distinct types of benchmark functions are deployed to investigate the SCWOA’s practicality and productivity: $f_{1}-f_{6}$ symbolizes unimodal, $f_{7}-f_{10}$ symbolizes multimodal, and $f_{11}-f_{16}$ symbolizes fixed-dimension multimodal. Table 1 summarizes the benchmark functions.

Table 2 summarizes each methodology’s parameters; these values are extracted from the initially published paper and symbolize representative empirical solutions.

For each methodology, the population magnitude symbolizes 50, the highest iteration symbolizes 1000, and the standalone operation symbolizes 30. Best, Worst, Mean, and Std are the optimal value, worst value, mean value, and standard deviation. The standard deviation ascertains the ranking.

Table 3 summarizes the simulation results of unimodal functions. For $f_{1}$, $f_{2}$, $f_{3}$, and $f_{4}$, the SCWOA establishes the international optimal solutions. The SCWOA’s Best, Worst, Mean, and Standard are consistently zero. The computerized solutions of the SCWOA are substantially more accurate than those of the fundamental WOA. The computational results of the SCWOA are more productive than those of the BA, CapSA, MFO, MVO, SAO, MDWA, and WOA, and the SCWOA exhibits great computational productivity and long-term durability to avert premature convergence and establish international optimal solutions. The SCWOA is ranked the highest and maintains outstanding durability and dependability. For $f_{5}$, the nonlinear strategy enhances the global detection ability and accelerates the convergence efficiency. The SCA constructed on the oscillation attributes of the cosine and sine coefficients in mathematics is a stochastic optimization methodology. The SCA upgrades population variety, amplifies the search region, and accelerates international investigation and regional extraction. The Best of the SCWOA is superior to that of the MVO and WOA but inferior to the BA, CapSA, MFO, SAO, and MDWA. The Worst and Mean of the SCWOA are more productive than those of the BA, MFO, MVO, and MDWA. The SCWOA is ranked fourth and has a lesser Standard than the BA, MFO, MVO, and MDWA. For $f_{6}$, the SCWOA integrates the SCA’s mathematical oscillation attributes with the nonlinear strategy to enhance the global optimization ability. The SCWOA utilizes exploration and exploitation to avoid premature convergence and enhance the optimization efficiency and exhibits exceptional sustainability and superiority to accelerate the leader whale’s location revision and improve the convergence precision. The computerized results of the SCWOA are more trustworthy than those of other competitive methodologies and are marginally more extraordinary than those of the WOA. The SCWOA integrates the WOA’s bubble-net scavenging pattern and SCA’s mathematical oscillation attributes to elevate population variety and maximize the operational area. The SCWOA exhibits fantastic durability and adaptability to strengthen investigation and extraction.

Table 4 summarizes the simulation results of multimodal functions. For $f_{7}$, the SCWOA, WOA, and CapSA establish the international optimal solutions, and their computerized solutions are all consistently zero. The simulation results of the SCWOA are superior to those of BA, MFO, MVO, SAO, and MDWA, and the SCWOA features’ attractive sustainability and authenticity promote convergence acceleration and elevate computational effectiveness. For $f_{8}$, the Best, Worst, and Mean of the SCWOA are all in the precise same order of magnitude; their computerized solutions remain comparable. The SCWOA’s computational results convey a minor enhancement over WOA. With the lowest Standard and highest ranking, the SCWOA highlights superiority and practicality in recognizing the more accurately generated solution. For $f_{9}$, the SCWOA and CapSA accomplish the most advantageous optimal solutions. The computerized solutions of the SCWOA are more accurate than the BA, MFO, MVO, SAO, MDWA, and WOA, and the SCWOA exhibited extraordinary superiority and adaptability to widen the investigation zone and accomplish more substantial conversion precision. For $f_{10}$, the computerized solutions of the SCWOA are inferior to those of the BA, CapSA, MFO, MDWA, and WOA but superior to those of the MVO and SAO. The SCWOA integrates investigation and extraction to inhibit premature convergence and exhibits remarkable superiority and adaptability to attain the desirable optimal solutions.

Table 5 summarizes the simulation results of fixed-dimension multimodal functions. For $f_{11}$, each methodology establishes the international optimal solutions. The computerized results of the SCWOA are more favorable than those of the BA, MFO, SAO, MDWA, and WOA, but they are inferior to those of the CapSA and MVO. The SCWOA is listed third and has solid reliability and dependability. For $f_{12}$, the SCWOA and BA establish the international optimal solutions. The computational results of the SCWOA have steadily improved. The computerized solutions of the SCWOA are the most outstanding. For $f_{13}$, all methodologies except SAO establish the international optimal solutions, and the automated results of the CapSA, MDWA, and SCWOA are identical. The computerized results of the SCWOA are more productive than those of the BA, MFO, MVO, SAO, and WOA. For $f_{14}$, all methodologies except SAO establish the international optimal solutions; the computational results of the SCWOA are more favorable than those of the BA, MFO, MDWA, and WOA. The SCWOA is identified fourth. For $f_{15}$, all methodologies except SAO establish the international optimal solutions, and the computational results of the CapSA, MDWA, and SCWOA are superior to those of BA, MFO, MVO, SAO, and WOA. For $f_{16}$, the SCWOA determines the international optimal solutions; the SCWOA’s Best, Worst, Mean, and Standard are all consistently zero. The computerized solutions of the SCWOA are superior to those of other competitive methodologies. The SCWOA exhibits certain practicability and superiority to recognize the quicker integration velocity and greater estimation precision and integrate global investigation and local extraction to acquire the most appropriate optimal solutions.

The Wilcoxon rank-sum explores the disparity between the SCWOA and competitive methodologies [30]. p<0.05 symbolizes the extraordinary disparity, p≥0.05 symbolizes no noteworthy disparity, and N/A symbolizes “not applicable”. Table 6 summarizes the results of the *p*-value Wilcoxon rank-sum.

**Figure 2:**
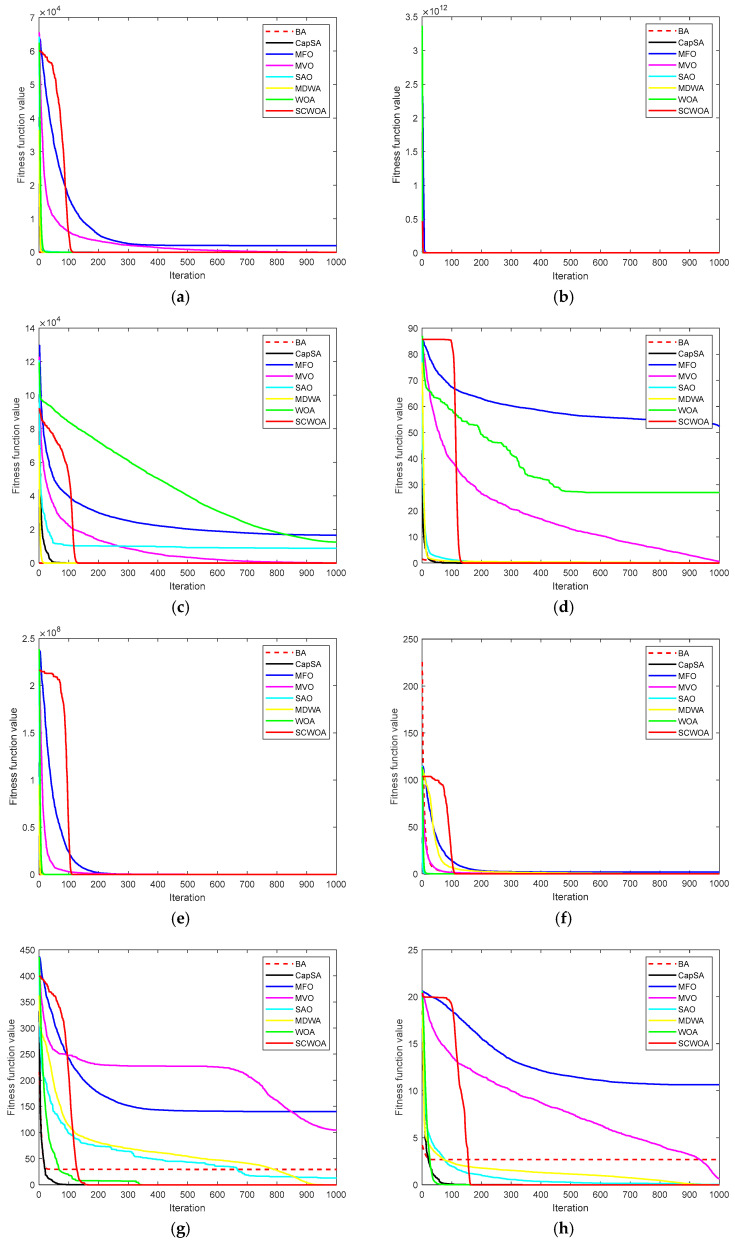

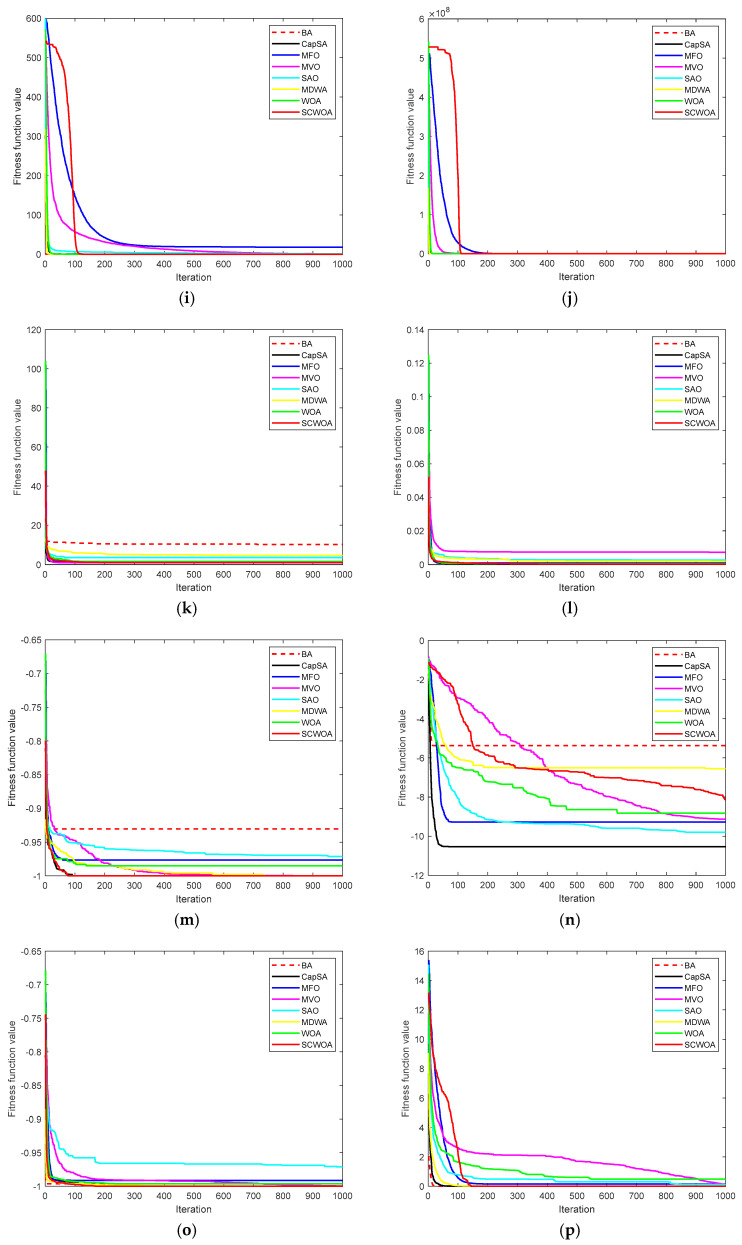
Convergence arcs of competitive methodologies. (**a**) $f_{1}$. (**b**) $f_{2}$. (**c**) $f_{3}$. (**d**) $f_{4}$. (**e**) $f_{5}$. (**f**) $f_{6}$. (**g**) $f_{7}$. (**h**) $f_{8}$. (**i**) $f_{9}$. (**j**) $f_{10}$. (**k**) $f_{11}$. (**l**) $f_{12}$. (**m**) $f_{13}$. (**n**) $f_{14}$. (**o**) $f_{15}$. (**p**) $f_{16}$.

Figure 2 portrays the convergence arcs of competitive methodologies. The convergence arcs illustrate the competitive methodology’s convergence productivity and computation sustainability unambiguously and objectively. The SCWOA exhibits outstanding superiority and practicability, as shown by more straightforward convergence velocity and more excellent computation reliability. For $f_{1}-f_{6}$, the computerized results of the SCWOA are superior to the BA, CapSA, MFO, MVO, SAO, MDWA, and WOA. The SCWOA’s automated solutions are substantially more accurate than the fundamental WOA ones. The computational results of the SCWOA are more productive than those of other competitive methodologies. The SCWOA receives a comparatively elevated rank, maintaining fantastic sustainability and dependability. For $f_{7}-f_{10}$, the SCWOA amalgamates international investigation and regional extraction to recognize the more accurate estimated solutions. The SCWOA encounters lower analytical accuracy and greater convergence productivity. For $f_{11}-f_{16}$, the SCWOA establishes the international optimal solutions, and the SCWOA delivers certain practicability and superiority to convey the most accurate estimated solutions. The analytical productivity of the SCWOA is superior to that of the BA, CapSA, MFO, MVO, SAO, MDWA, and WOA. The SCWOA exhibits outstanding consistency and robustness to avert premature convergence and cultivate the most accurate optimal solutions.

**Figure 3:**
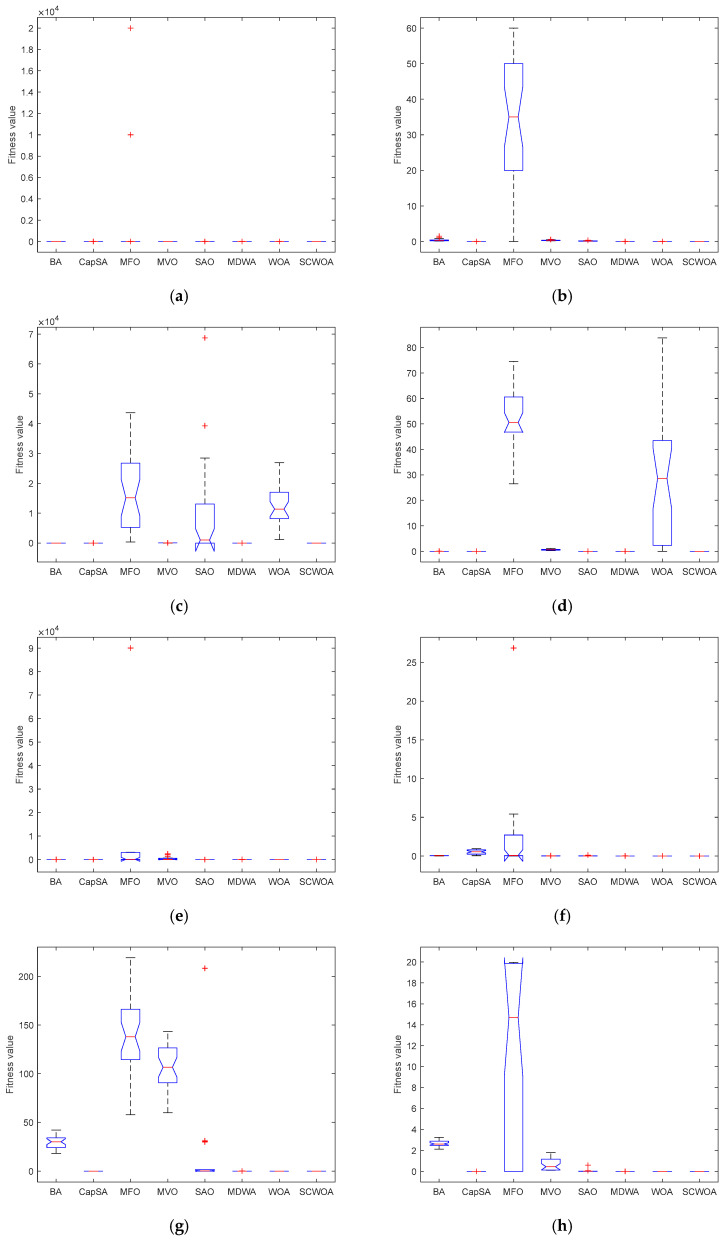

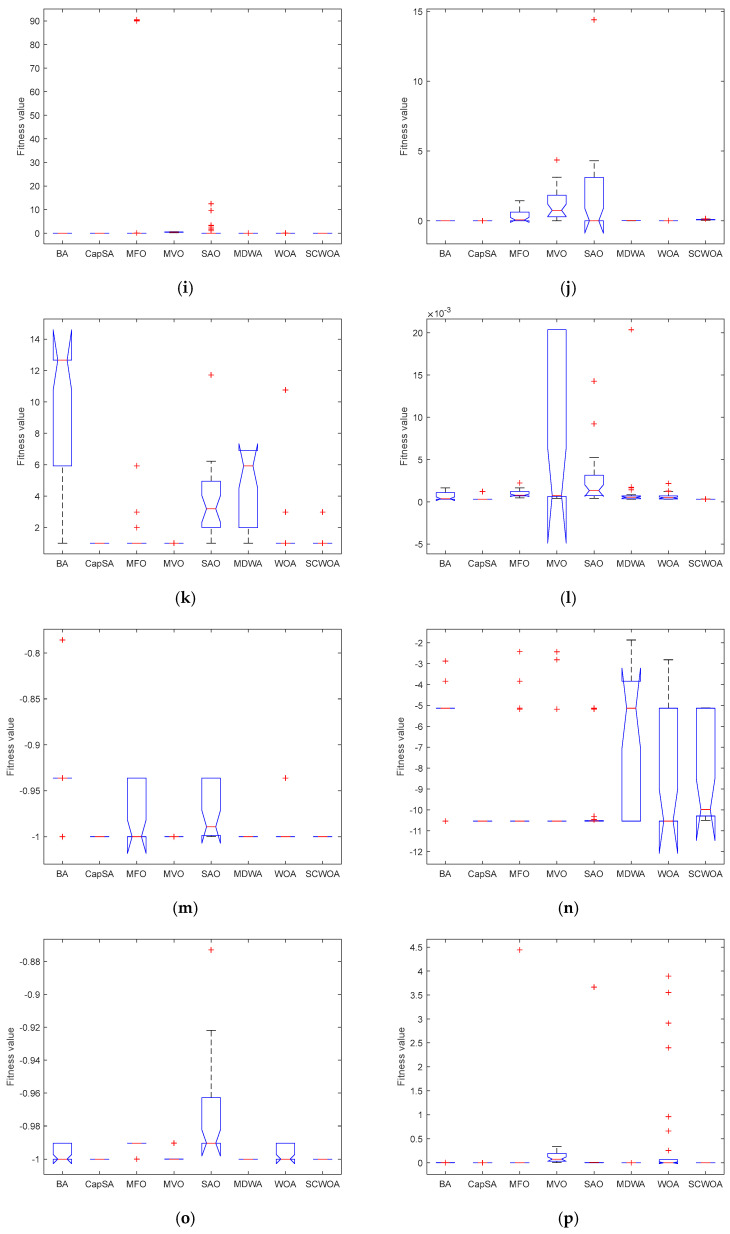
ANOVA tests of competitive methodologies. (**a**) $f_{1}$. (**b**) $f_{2}$. (**c**) $f_{3}$. (**d**) $f_{4}$. (**e**) $f_{5}$. (**f**) $f_{6}$. (**g**) $f_{7}$. (**h**) $f_{8}$. (**i**) $f_{9}$. (**j**) $f_{10}$. (**k**) $f_{11}$. (**l**) $f_{12}$. (**m**) $f_{13}$. (**n**) $f_{14}$. (**o**) $f_{15}$. (**p**) $f_{16}$.

Figure 3 portrays the ANOVA of competitive methodologies. The standard deviation shows the competitive methodology’s reliability and long-term stability objectively and straightforwardly. The less extensive standard deviation demonstrates remarkable dependability and trustworthiness. For $f_{1}-f_{6}$, the computational results of the SCWOA have been extensively facilitated, and the standard deviation and ranking of the SCWOA are more favorable than those of other competitive technologies. The SCWOA demonstrates fantastic durability and adaptability to measure the potential values. For $f_{7}-f_{10}$, the SCWOA exemplifies superior convergence effectiveness and greater computation profitability. The SCWOA exhibits integrate investigation and extraction to avert anticipation stalemate and identify the highest-quality alternative solution. For $f_{11}-f_{16}$, the SCWOA delivers the international optimal solutions. The SCWOA’s computerized solutions have been substantially upgraded compared to those of the WOA. The standard deviation of the SCWOA is greater than that of other competitive methodologies, and the SCWOA gains considerable superiority and adaptability to maintain feasibility and stability. The SCWOA utilizes sine and cosine fluctuation characteristics to prevent preterm convergence and discover the most advantageous optimal solutions.

### 4.3. SCWOA for Addressing Engineering Design

The SCWOA is designed to recognize the engineering design to validate the comprehensiveness and practicality: three-bar truss design [31], tubular column design [32], speed reducer design [33], piston lever design [34], tension/compression spring design [35], welded beam design [36], gear train design [37], and car side impact design [38].

#### 4.3.1. Three-Bar Truss Design

**Figure 4:**
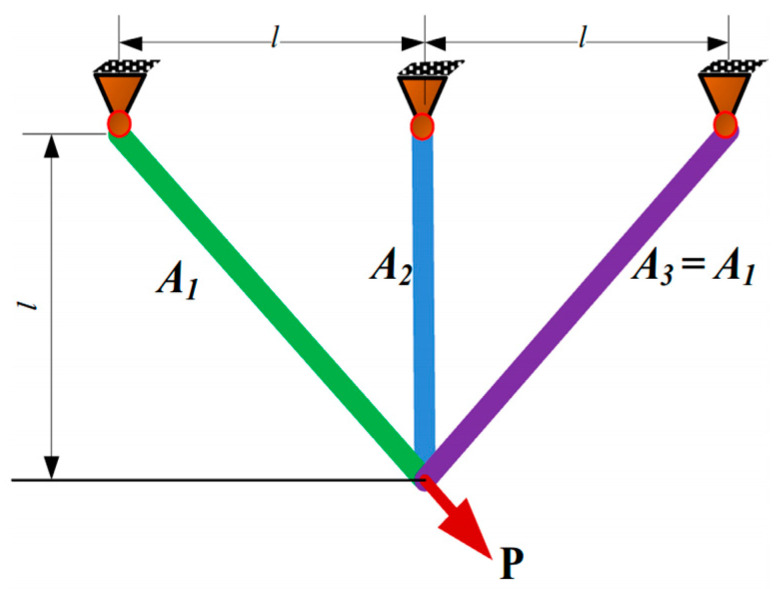
Three-bar truss design.

The predominant intention is to lessen the aggregate weight, as portrayed in Figure 4. There are two decision elements: the cross-sectional areas with $A_{1}$ and $A_{2}$.

Consider

(18) $$x=[x_{1}\;\;\;x_{2}]=[A_{1}\;\;\;A_{2}]$$

Minimize

(19) $$f(x)=(2\sqrt{2}\cdot x_{1}+x_{2})\cdot l$$

Subject to

(20) $$g_{1}(x)=\frac{\sqrt{2}\cdot x_{1}+x_{2}}{\sqrt{2}\cdot x_{1}^{2}+2\cdot x_{1}\cdot x_{2}}P-\sigma \leq 0$$

(21) $$g_{2}(x)=\frac{x_{2}}{\sqrt{2}\cdot x_{2}+2\cdot x_{1}\cdot x_{2}}P-\sigma \leq 0$$

(22) $$g_{3}(x)=\frac{1}{\sqrt{2}\cdot x_{2}+x_{1}}P-\sigma \leq 0$$

(23) $$l=100cm,\;\;\;\;\;\;P=2kN/cm^{2},\;\;\;\;\;\;\sigma =2kN/cm^{2}$$

Variable range

(24) $$0\leq x_{1},x_{2}\leq 1$$

Table 7 summarizes the comparison results of the three-bar truss design. The SCWOA maintains tremendous reliability and remarkable computational productivity to yield optimum established solutions. The optimal solution is manufactured via the SCWOA at design elements of 0.788674 and 0.408234, with the least productive cost of 263.895843.

#### 4.3.2. Tubular Column Design

**Figure 5:**
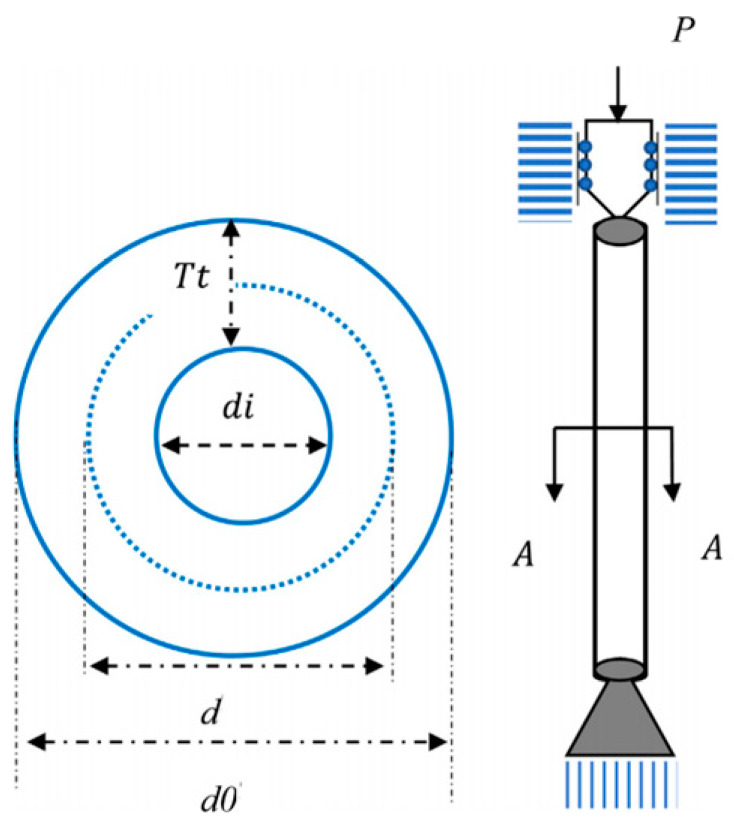
Tubular column design.

The predominant intention is to lessen the aggregate cost of installation and equipment, as portrayed in Figure 5. There are two decision elements: column width (d) and tube layer (t).

Consider

(25) $$x=[x_{1}\;\;\;x_{2}]=[d\;\;\;t]$$

Minimize

(26) $$f(x)=9.82\cdot x_{1}\cdot x_{2}+2x_{1}$$

Subject to

(27) $$g_{1}(x)=\frac{P}{\pi x_{1}\cdot x_{2}\cdot \sigma_{y}}-1\leq 0$$

(28) $$g_{2}(x)=\frac{8P\cdot L^{2}}{\pi^{3}\cdot E\cdot x_{1}\cdot x_{2}\cdot (x_{1}^{2}+x_{2}^{2})}-1\leq 0$$

(29) $$g_{3}(x)=\frac{2.0}{x_{1}}-1\leq 0$$

(30) $$g_{4}(x)=\frac{x_{1}}{14}-1\leq 0$$

(31) $$g_{5}(x)=\frac{0.2}{x_{2}-1}\leq 0$$

(32) $$g_{6}(x)=\frac{x_{2}}{0.8}-1\leq 0$$

(33) $$\sigma_{y}=500kgf/cm^{2},\;\;\;\;\;\;E=0.85\times 10^{6}kgf/cm^{2},\;\;\;\;\;\;P=2500kgf,\;\;\;\;\;\;L=250cm$$

Variable range

(34) $$2\leq x_{1}\leq 14,\;\;\;\;\;\;0.2\leq x_{2}\leq 0.8$$

Table 8 summarizes the comparison results of the tubular column design. The SCWOA accomplishes international investigation and regional extraction to expand population variety and provide appropriate solutions. The optimal solution is manufactured via the SCWOA at design elements of 5.5537 and 0.2502, with the least productive cost of 25.5346.

#### 4.3.3. Speed Reducer Design

**Figure 6:**
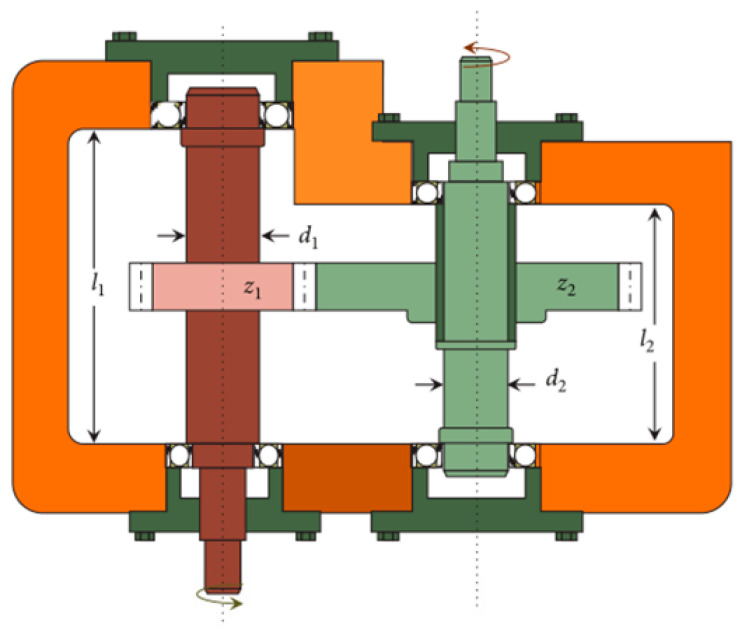
Speed reducer design.

The predominant intention is to lessen the aggregate weight, as portrayed in Figure 6. There are seven decision elements: face breadth (b), dental module (m), dental size (z), primary shaft distance ($l_{1}$), second shaft distance ($l_{2}$), primary shaft width ($d_{1}$), and second shaft width ($d_{2}$).

Consider

(35) $$x=[x_{1}\;\;\;x_{2}\;\;\;x_{3}\;\;\;x_{4}\;\;\;\;x_{5}\;\;\;\;x_{6}\;\;\;\;x_{7}]=[b\;\;\;m\;\;\;z\;\;\;\;l_{1}\;\;\;l_{2}\;\;\;d_{1}\;\;\;d_{2}]$$

Minimize

(36) $$\begin{array}{l} f(x)=0.7854\cdot x_{1}\cdot x_{2}^{2}(3.3333\cdot x_{3}^{2}+14.9334\cdot x_{3}-43.0934)\\ \;\;\;\;\;\;\;\;\;\;-1.508\cdot x_{1}\cdot (x_{6}^{2}+x_{7}^{2})+7.4777\cdot (x_{6}^{3}+x_{7}^{3})+0.7854\cdot (x_{4}\cdot x_{6}^{2}+x_{5}\cdot x_{7}^{2}) \end{array}$$

Subject to

(37) $$g_{1}(x)=\frac{27}{x_{1}\cdot x_{2}^{2}\cdot x_{3}^{2}}-1\leq 0$$

(38) $$g_{2}(x)=\frac{397.5}{x_{1}\cdot x_{2}^{2}\cdot x_{3}}-1\leq 0$$

(39) $$g_{3}(x)=\frac{1.93\cdot x_{4}^{3}}{x_{2}\cdot x_{6}^{4}\cdot x_{3}}-1\leq 0$$

(40) $$g_{4}(x)=\frac{1.93\cdot x_{5}^{3}}{x_{2}\cdot x_{7}^{5}\cdot x_{3}}-1\leq 0$$

(41) $$g_{5}(x)=\frac{[(745\cdot x_{4}/x_{2}\cdot x_{3}{)}^{2}+16.9\times 10^{6}{]}^{1/2}}{110\cdot x_{6}^{3}}-1\leq 0$$

(42) $$g_{6}(x)=\frac{[(745\cdot x_{5}/x_{2}\cdot x_{3}{)}^{2}+157.5\times 10^{6}{]}^{1/2}}{85\cdot x_{7}^{3}}-1\leq 0$$

(43) $$g_{7}(x)=\frac{x_{2}\cdot x_{3}}{40}-1\leq 0$$

(44) $$g_{8}(x)=\frac{5\cdot x_{2}}{x_{1}}-1\leq 0$$

(45) $$g_{9}(x)=\frac{x_{1}}{12\cdot x_{2}}-1\leq 0$$

(46) $$g_{10}(x)=\frac{1.5\cdot x_{6}+1.9}{x_{4}}-1\leq 0$$

(47) $$g_{11}(x)=\frac{1.1\cdot x_{7}+1.7}{x_{5}}-1\leq 0$$

Variable range

(48) $$2.6\leq x_{1}\leq 3.6,\;\;\;0.7\leq x_{2}\leq 0.8,\;\;\;17\leq x_{3}\leq 28,\;\;\;7.3\leq x_{4},x_{5}\leq 8.3,\;\;\;2.9\leq x_{6}\leq 3.9,\;\;\;5.0\leq x_{7}\leq 5.5$$

Table 9 summarizes the comparison results of the speed reducer design. The SCWOA utilizes the SCA’s mathematical oscillation attributes and the WOA’s bubble-net scavenging pattern to enhance the convergence accuracy. The SCWOA manufactures the optimal solution at design elements of 3.50228, 0.7, 17, 7.88793, 7.82363, 3.36347, and 5.29537 with the least productive cost of 3017.596.

#### 4.3.4. Piston Lever Design

**Figure 7:**
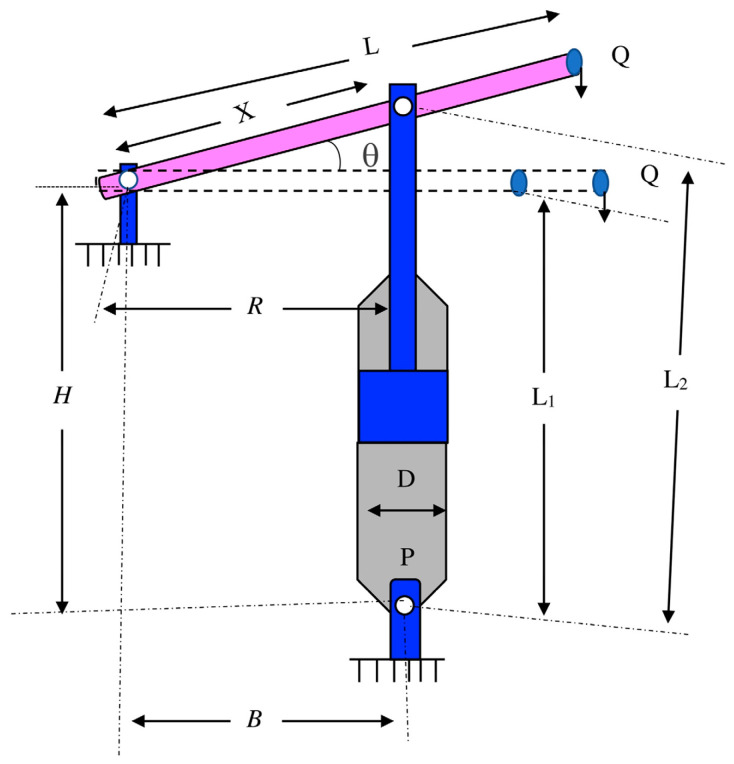
Piston lever design.

The predominant intention is to restrict the gasoline volume and maneuver the piston aspects if the piston lever is elevated from 0° to 45°, as portrayed in Figure 7. There are four decision elements: H, B, X, and D.

Consider

(49) $$x=[x_{1}\;\;\;x_{2}\;\;\;x_{3}\;\;\;x_{4}]=[H\;\;\;B\;\;\;D\;\;\;X]$$

Minimize

(50) $$f(x)=\frac{1}{4}\pi \cdot x_{3}^{2}(L_{2}-L_{1})$$

Subject to

(51) $$g_{1}(x)=Q\cdot L\cdot \mathrm{os}\theta -R\cdot F\leq 0$$

(52) $$g_{2}(x)=Q\cdot (L-x_{4})-M_{\max}\leq 0$$

(53) $$g_{3}(x)=\frac{6}{5}\cdot (L_{2}-L_{1})-L_{1}\leq 0$$

(54) $$g_{4}(x)=\frac{x_{3}}{2}-x_{2}\leq 0$$

(55) $$R=\frac{\left|-x_{4}\cdot (x_{4}\sin\theta +x_{1})+x_{1}\cdot (x_{2}-x_{4}\cos\theta )\right|}{\sqrt{{\left(x_{4}-x_{2}\right)}^{2}+x_{1}^{2}}}$$

(56) $$F=\frac{\pi \cdot P\cdot x_{3}^{2}}{4}$$

(57) $$L_{1}=\sqrt{{\left(x_{4}-x_{2}\right)}^{2}+x_{1}^{2}}$$

(58) $$L_{2}=\sqrt{{\left(x_{4}\cdot \sin\theta +x_{1}\right)}^{2}+{\left(x_{2}-x_{4}\cdot \cos\theta \right)}^{2}}$$

(59) $$\theta =45{}^{\circ},\;\;\;Q=10,000lbs,\;\;\;L=240in,\;\;\;M_{\max}=1.8\times 10^{6}lbs\;\;in,\;\;\;P=1500psi$$

Variable range

(60) $$0.05\leq x_{1},x_{2},x_{4}\leq 500,\;\;\;\;\;\;0.05\leq x_{3}\leq 120$$

Table 10 summarizes the comparison results of the piston lever design. The SCWOA delivers convincing practicability and superiority to strengthen solution quality and computational effectiveness. The optimal solution is manufactured via the SCWOA at design elements of 0.05, 0.138542, 120, and 4.116025, with the least productive cost of 4.6827.

#### 4.3.5. Tension/Compression Spring Design

**Figure 8:**
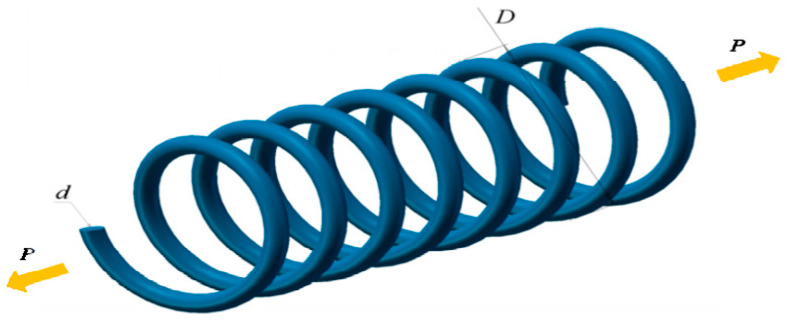
Tension/compression spring design.

The predominant intention is to lessen the aggregate weight, as portrayed in Figure 8. There are three decision elements: line diameter (d), spring diameter (D), and activated coil size (N).

Consider

(61) $$x=[x_{1}\;\;\;x_{2}\;\;\;x_{3}\;]=[d\;\;\;D\;\;\;N]\;\;\;$$

Minimize

(62) $$f(x)=(x_{3}+2)\cdot x_{2}\cdot x_{1}^{2}$$

Subject to

(63) $$g_{1}(x)=1-\frac{x_{2}^{3}\cdot x_{3}}{71,785\cdot x_{1}^{4}}\leq 0$$

(64) $$g_{2}(x)=\frac{4\cdot x_{2}^{2}-x_{1}\cdot x_{2}}{12,566\cdot (x_{2}\cdot x_{1}^{3}-x_{1}^{4})}+\frac{1}{5108\cdot x_{1}^{2}}\leq 0$$

(65) $$g_{3}(x)=1-\frac{140.45\cdot x_{1}}{x_{2}^{2}\cdot x_{3}}\leq 0$$

(66) $$g_{4}(x)=\frac{x_{1}+x_{2}}{1.5}-1\leq 0$$

Variable range

(67) $$0.05\leq x_{1}\leq 2,\;\;\;\;\;\;0.25\leq x_{2}\leq 1.3,\;\;\;\;\;\;2\leq x_{3}\leq 15$$

Table 11 summarizes the comparison results of the tension/compression spring design. The SCWOA depicts outstanding adaptability and fantastic computational accuracy to disrupt preterm convergence and establish the most advantageous optimal solutions. The optimal solution is manufactured via the SCWOA at design elements of 0.054627, 0.325243, and 11.654662, with the least productive cost of 0.0126653.

#### 4.3.6. Welded Beam Design

**Figure 9:**
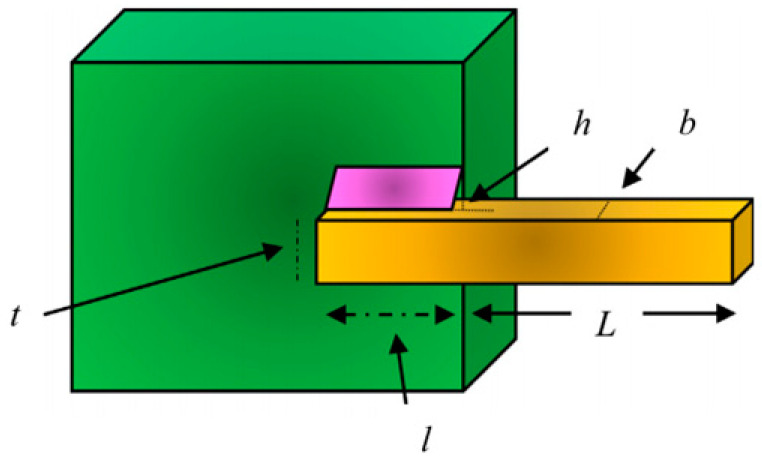
Welded beam design.

The predominant intention is to lessen the aggregate cost, as portrayed in Figure 9. There are four decision elements: weld height (h), clamped bar depth (l), bar width (t), and bar height (b).

Consider

(68) $$x=[x_{1}\;\;\;x_{2}\;\;\;x_{3}\;\;\;x_{4}]=[h\;\;\;l\;\;\;t\;\;\;b]$$

Minimize

(69) $$f(x)=1.10471\cdot x_{1}^{2}\cdot x_{2}+0.04811\cdot x_{3}\cdot x_{4}(14.0+x_{2})$$

Subject to

(70) $$g_{1}(x)=\tau (x)-\tau_{\max}\leq 0$$

(71) $$g_{2}(x)=\sigma (x)-\sigma_{\max}\leq 0$$

(72) $$g_{3}(x)=\delta (x)-\delta_{\max}\leq 0$$

(73) $$g_{4}(x)=x_{1}-x_{4}\leq 0$$

(74) $$g_{5}(x)=P-P_{c}(x)\leq 0$$

(75) $$g_{6}(x)=0.125-x_{1}\leq 0$$

(76) $$g_{7}(x)=1.10471\cdot x_{1}^{2}+0.04811\cdot x_{3}\cdot x_{4}(14.0+x_{2})-5.0\leq 0$$

(77) $$\tau (x)=\sqrt{{\left(\tau^{\prime }\right)}^{2}+2\tau^{\prime }\tau^{\prime \prime }\frac{x_{2}}{2R}+{\left(\tau^{\prime \prime }\right)}^{2}}$$

(78) $$\tau^{\prime }=\frac{P}{\sqrt{2}x_{1}x_{2}},\;\;\;\;\;\;\tau^{\prime \prime }=\frac{MP}{J},\;\;\;\;\;\;M=P(L+\frac{x_{2}}{2})$$

(79) $$R=\sqrt{\frac{x_{2}^{2}}{4}+{\left(\frac{x_{1}+x_{3}}{2}\right)}^{2}}$$

(80) $$J=2\left\{\sqrt{2}\cdot x_{1}\cdot x_{2}\left[\frac{x_{2}^{2}}{4}+{\left(\frac{x_{1}+x_{3}}{2}\right)}^{2}\right]\right\}$$

(81) $$\sigma (x)=\frac{6P\cdot L}{x_{4}\cdot x_{3}^{2}},\;\;\;\;\;\;\delta (x)=\frac{6P\cdot L^{3}}{E\cdot x_{3}^{2}\cdot x_{4}}$$

(82) $$P_{c}(x)=\frac{4.103E\sqrt{\frac{x_{3}^{2}\cdot x_{4}^{6}}{36}}}{L^{2}}\left(1-\frac{x_{3}}{2L}\sqrt{\frac{E}{4G}}\right)$$

(83) $$P=6000lb,\;\;\;\;\;\;L=14in,\;\;\;\;\;\;\delta_{\max}=0.25in$$

(84) $$E=30\times 10^{6}psi,\;\;\;\;\;\;G=12\times 10^{6}psi$$

(85) $$\tau_{\max}=13,600psi,\;\;\;\;\;\;\sigma =30,000psi$$

Variable range

(86) $$0.1\leq x_{1},x_{4}\leq 2,\;\;\;\;\;\;0.1\leq x_{2},x_{3}\leq 10$$

Table 12 summarizes the comparison results of the welded beam design. The SCWOA exhibits unparalleled sustainability and parallelism to facilitate the investigation zone and recognize accurate optimal solutions. The SCWOA manufactures the optimal solution at design elements of 0.205657, 3.251177, 9.039105, and 0.205468 with the least productive cost of 1.69682.

#### 4.3.7. Gear Train Design

**Figure 10:**
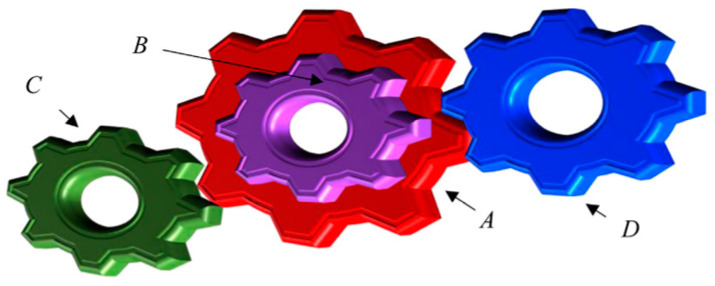
Gear train design.

The predominant intention is to lessen the gear ratio’s cost and ascertain the greatest tooth size, as portrayed in Figure 10. There are four decision elements: gear teeth’s quantity $n_{A}$, $n_{B}$, $n_{C}$, and $n_{D}$.

Consider

(87) $$x=[x_{1}\;\;\;x_{2}\;\;\;x_{3}\;\;\;x_{4}]=[n_{A}\;\;\;n_{B}\;\;\;n_{C}\;\;\;n_{D}]$$

Minimize

(88) $$f(x)={\left(\frac{1}{6.931}-\frac{x_{3}\cdot x_{2}}{x_{1}\cdot x_{4}}\right)}^{2}$$

Variable range

(89) $$12\leq x_{i}\leq 60,\;\;\;i=1,2,\ldots ,4$$

Table 13 summarizes the comparison results of the gear train design. The SCWOA exhibits trustworthy international investigation and localized extraction to acquire greater computation precision and quicker processing velocity. The optimal solution is manufactured via the SCWOA at design elements of 51, 33, 17, and 53, with the least productive cost of 2.6574 × 10^−18^.

#### 4.3.8. Car Side Impact Design

**Figure 11:**
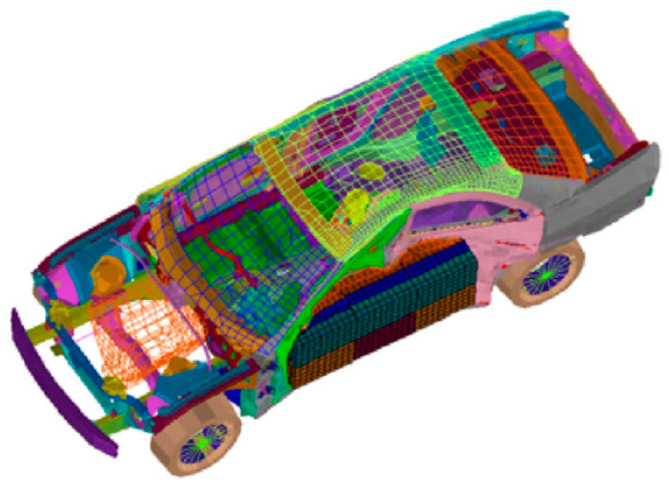
Car side impact design.

The predominant intention is to lessen the aggregate car’s weight, as portrayed in Figure 11. There are eleven decision elements: inner B-pillar height ($x_{1}$), B-pillar fortification ($x_{2}$), interior floor edge ($x_{3}$), cross segments ($x_{4}$), door pillar ($x_{5}$), consolidated door beltline ($x_{6}$), roof panel ($x_{7}$), inner B-pillar substrates ($x_{8}$), interior floor edge ($x_{9}$), barrier width ($x_{10}$), and batting location ($x_{11}$).

Consider

(90) $$x=[x_{1}\;\;\;x_{2}\;\;\;x_{3}\;\;\;x_{4}\;\;\;x_{5}\;\;\;x_{6}\;\;\;x_{7}\;\;\;x_{8}\;\;\;x_{9}\;\;\;x_{10}\;\;\;x_{11}]$$

Minimize

(91) $$f(x)=1.98+4.90\cdot x_{1}+6.67\cdot x_{2}+6.98\cdot x_{3}+4.01\cdot x_{4}+1.78\cdot x_{5}+2.73\cdot x_{7}$$

Subject to

(92) $$\begin{array}{l} g_{1}(x)=1.16-0.3717\cdot x_{2}\cdot x_{4}-0.00931\cdot x_{2}\cdot x_{10}-0.484\cdot x_{3}\cdot x_{9}\\ \;\;\;\;\;\;\;\;\;\;\;\;+0.01343\cdot x_{6}\cdot x_{10}\leq 1 \end{array}$$

(93) $$\begin{array}{l} g_{2}(x)=0.261-0.0159\cdot x_{1}\cdot x_{2}-0.188\cdot x_{1}\cdot x_{8}-0.019\cdot x_{2}\cdot x_{7}\\ \;\;\;\;\;\;\;\;\;\;\;\;+0.0144\cdot x_{3}\cdot x_{5}+0.0008757\cdot x_{5}\cdot x_{10}+0.080405\cdot x_{6}\cdot x_{9}\\ \;\;\;\;\;\;\;\;\;\;\;\;+0.00139\cdot x_{8}\cdot x_{11}+0.00001575\cdot x_{10}\cdot x_{11}\leq 0.32 \end{array}$$

(94) $$\begin{array}{l} g_{3}(x)=0.214+0.00817\cdot x_{5}-0.131\cdot x_{1}\cdot x_{8}-0.0704\cdot x_{1}\cdot x_{9}\\ \;\;\;\;\;\;\;\;\;\;\;\;+0.03099\cdot x_{2}\cdot x_{6}-0.018\cdot x_{2}\cdot x_{7}+0.0208\cdot x_{3}\cdot x_{8}+0.121\cdot x_{3}\cdot x_{9}\\ \;\;\;\;\;\;\;\;\;\;\;\;-0.00364\cdot x_{5}\cdot x_{6}+0.0007715\cdot x_{5}\cdot x_{10}-0.000535\cdot x_{6}\cdot x_{10}\\ \;\;\;\;\;\;\;\;\;\;\;\;+0.00121\cdot x_{8}\cdot x_{11}\leq 0.32 \end{array}$$

(95) $$\begin{array}{l} g_{4}(x)=0.074-0.061\cdot x_{2}-0.163\cdot x_{3}\cdot x_{8}+0.001232\cdot x_{3}\cdot x_{10}\\ \;\;\;\;\;\;\;\;\;\;\;\;-0.166\cdot x_{7}\cdot x_{9}+0.227\cdot x_{2}^{2}\leq 0.32 \end{array}$$

(96) $$\begin{array}{l} g_{5}(x)=28.98+3.818\cdot x_{3}-4.2\cdot x_{1}\cdot x_{2}+0.0207\cdot x_{5}\cdot x_{10}+6.63\cdot x_{6}\cdot x_{9}\\ \;\;\;\;\;\;\;\;\;\;\;\;-7.7\cdot x_{7}\cdot x_{8}+0.32\cdot x_{9}\cdot x_{10}\leq 32 \end{array}$$

(97) $$\begin{array}{l} g_{6}(x)=33.86+2.95\cdot x_{3}+0.1792\cdot x_{10}-5.057\cdot x_{1}\cdot x_{2}-11.0\cdot x_{2}\cdot x_{8}\\ \;\;\;\;\;\;\;\;\;\;\;\;-0.0215\cdot x_{5}\cdot x_{10}-9.98\cdot x_{7}\cdot x_{8}+22.0\cdot x_{8}\cdot x_{9}\leq 32 \end{array}$$

(98) $$g_{7}(x)=46.36-9.9\cdot x_{2}-12.9\cdot x_{1}\cdot x_{8}+0.1107\cdot x_{3}\cdot x_{10}\leq 32$$

(99) $$\begin{array}{l} g_{8}(x)=4.72-0.5\cdot x_{4}-0.19\cdot x_{2}\cdot x_{3}-0.0122\cdot x_{4}\cdot x_{10}\\ \;\;\;\;\;\;\;\;\;\;\;\;+0.009325\cdot x_{6}\cdot x_{10}+0.000191\cdot x_{11}^{2}\leq 4 \end{array}$$

(100) $$\begin{array}{l} g_{9}(x)=10.58-0.674\cdot x_{1}\cdot x_{2}-1.95\cdot x_{2}\cdot x_{8}+0.02054\cdot x_{3}\cdot x_{10}\\ \;\;\;\;\;\;\;\;\;\;\;\;-0.0198\cdot x_{4}\cdot x_{10}+0.028\cdot x_{6}\cdot x_{10}\leq 9.9 \end{array}$$

(101) $$\begin{array}{l} g_{10}(x)=16.45-0.489\cdot x_{3}\cdot x_{7}-0.843\cdot x_{5}\cdot x_{6}+0.0432\cdot x_{9}\cdot x_{10}\\ \;\;\;\;\;\;\;\;\;\;\;\;-0.0556\cdot x_{9}\cdot x_{11}-0.000786\cdot x_{11}^{2}\leq 15.7 \end{array}$$

Variable range

(102) $$0.5\leq x_{1}-x_{7}\leq 1.5,\;\;\;\;\;\;x_{8},x_{9}\in (0.192,0.345),\;\;\;\;\;\;-30\leq x_{10},x_{11}\leq 30$$

Table 14 summarizes the comparison results of the three-bar truss design. The SCWOA utilizes SCA’s mathematical oscillation attributes to elevate population variety, enlarge investigation region, and identify the theoretically appropriate values. The SCWOA manufactures the optimal solution at design elements of 0.5, 1.11643, 0.5, 1.30178, 0.5, 1.5, 0.5, 0.345, 0.192, −19.48754, and −0.00453 with the least productive cost of 22.84278.

## 5. Conclusions and Future Investigation

This paper presents the SCWOA to recognize the benchmark functions and engineering designs, and the ultimate intention is to estimate the function’s international optimal value and the design’s appropriate cost. The SCA executes the mathematical oscillation fluctuation to undergo expansive investigations and ascertain the most relevant computational solution, which appropriately eliminates the WOA’s majority limitations of restricted precision, sluggish convergence acceleration, insufficient population variety, easy premature convergence, and restricted operational efficiency. The SCWOA exhibits exceptional sustainability and superiority to accelerate the leader whale’s location revision and strengthen calculational productiveness. The computerized solutions and appropriate cost of the SCWOA are substantially superior to those of the BA, CapSA, MFO, MVO, SAO, MDWA, and WOA, and the SCWOA illustrates fantastic adaptability and favorable computational accuracy to enlarge the identification scope and ascertain the most accurate optimal solution. The experimental results emphasize that the SCWOA not only utilizes universal investigation and regional extraction to avert premature convergence and strengthen optimization achievements but also maintains courageous practicability and superiority to pursue faster convergence velocity, greater computation precision, robust adaptability, and robustness.

In future research, we will rely on the Anhui Provincial Understory Crop Intelligent Equipment Engineering Research Center platform. The SCGJO will study specialty crops in the Dabie Mountains (dendrobium, tea-oil tree, and Chinese herbal medicine), such as intelligent detection of agricultural and forestry crop objects, research and development of special agricultural equipment, and the agricultural Internet of Things.

## Figures and Tables

**Table 1:** Benchmark functions.

Benchmark Test Functions	Dim	Range	$f_{\min}$
$f_{1}=\sum_{i=1}^{n}x_{i}^{2}$	30	[−100, 100]	0
$f_{2}(x)=\sum_{i=1}^{n}\left|x_{i}\right|+\prod_{i=1}^{n}\left|x_{i}\right|$	30	[−10, 10]	0
$f_{3}(x)=\sum_{i=1}^{n}{\left(\sum_{j=1}^{i}x_{j}\right)}^{2}$	30	[−100, 100]	0
$f_{4}(x)=\max_{i}\{|x_{i}|,1\leq i\leq n\}$	30	[−100, 100]	0
$f_{5}(x)=\sum_{i=1}^{n-1}\left[100{\left(x_{i+1}-x_{i}^{2}\right)}^{2}+{\left(x_{i}-1\right)}^{2}\right]$	30	[−30, 30]	0
$f_{6}(x)=\sum_{i=1}^{n}ix_{i}^{4}+random[0,1)$	30	[−1.28, 1.28]	0
$f_{7}(x)=\sum_{i=1}^{n}\left[x_{i}^{2}-10\cos(2\pi x_{i})+10\right]$	30	[−5.12, 5.12]	0
$\begin{array}{l} f_{8}(x)=-20\exp\left(-0.2\sqrt{\frac{1}{n}\sum_{i=1}^{n}x_{i}^{2}}-\exp\left(\frac{1}{n}\sum_{i=1}^{n}\cos2\pi x_{i}\right)\right)\\ +20+e \end{array}$	30	[−32, 32]	0
$f_{9}(x)=\frac{1}{4000}\sum_{i=1}^{n}\left(x_{i}^{2}\right)-\prod_{i=1}^{n}\cos\left(\frac{x_{i}}{\sqrt{i}}\right)+1$	30	[−600, 600]	0
$\begin{array}{l} f_{10}(x)=\frac{\pi }{n}\left\{10\sin^{2}(\pi y_{1})+\sum_{i=1}^{n-1}\begin{array}{l} {\left(y-1\right)}^{2}[1+10\sin^{2}(\pi y_{1})]\\ +{\left(y_{n}-1\right)}^{2} \end{array}\right\}\\ +\sum_{i=1}^{n}u(x_{i},10,100,4)\\ y_{i}=1+\frac{x_{i}+1}{4}\\ u(x_{i},a,k,m)=\left\{\begin{array}{l} k{\left(x_{i}-a\right)}^{m},x^{i}>a\\ 0,-a\leq x_{i}\leq a\\ k{\left(-x_{i}-z\right)}^{m},x_{i}<a \end{array}\right. \end{array}$	30	[−50, 50]	0
$f_{11}(x)={\left(\frac{1}{500}+\sum_{j=1}^{25}\frac{1}{j+\sum_{i=1}^{2}{\left(x_{i}-a_{ij}\right)}^{6}}\right)}^{-1}$	2	[−65, 65]	0.998
$f_{12}(x)=\sum_{i=1}^{11}{\left[a_{i}-\frac{x_{1}(b_{i}^{2}+b_{i}x_{2})}{b_{i}^{2}+b_{i}x_{3}+x_{4}}\right]}^{2}$	4	[−5, 5]	0.000308
$f_{13}(x)=-\frac{1+\cos(12\sqrt{x_{1}^{2}+x_{2}^{2}})}{0.5(x_{1}^{2}+x_{2}^{2})+2}$	2	[−5.12, 5.12]	−1
$f_{14}(x)=-\sum_{i=1}^{10}{\left[(x-a_{i}){\left(x-a_{i}\right)}^{T}+c_{i}\right]}^{-1}$	4	[0, 10]	−10.5364
$f_{15}(x)=0.5+\frac{\sin^{2}\left(\sqrt{x_{1}^{2}+x_{2}^{2}}\right)-0.5}{{\left(1+0.001(x_{1}^{2}+x_{2}^{2})\right)}^{2}}$	2	[−100, 100]	−1
$f_{16}(x)=\sum_{i=1}^{n}x_{i}\sin(x_{i})+0.1x_{i}$	10	[−10, 10]	0

**Table 2:** Initial parameters of each methodology.

Methodology	Parameter	Value
BA	Pulse frequency f	[0, 2]
	Echo loudness A	0.25
	Decreasing coefficient γ	0.5
CapSA	Disordered solution ε	[0, 1]
	Balance probability $P_{bf}$	0.7
	Gravitational force g	9.81
	Disordered solution r	[0, 1]
	Solution $\beta_{0}$	2
	Solution $\beta_{1}$	21
	Solution $\beta_{2}$	2
	Inertia coefficient ρ	0.7
MFO	Constant b	1
	Disordered solution t	[−1, 1]
	Disordered solution r	[−2, −1]
MVO	Disordered solution $r_{1}$	[0, 1]
	Disordered solution $r_{2}$	[0, 1]
	Disordered solution $r_{3}$	[0, 1]
	Disordered solution $r_{4}$	[0, 1]
	Exploitation accuracy p	6
	Minimum probability WEP_Min	0.2
	Maximum probability WEP_Max	1
SAO	Disordered solution $r_{0}$	(0, 1]
	Smell constant k	0.6
	Temperature of smell molecules T	0.95
	Mass of smell molecules m	0.9
	Disordered solution $r_{1}$	(0, 1]
	Disordered solution $r_{2}$	(0, 1]
	Disordered solution $r_{3}$	(0, 1]
	Disordered solution $r_{4}$	(0, 1]
MDWA	Constant $a_{\max}$	1
	Constant $a_{\min}$	0
WOA	Disordered solution $r_{1}$	[0, 1]
	Disordered solution $r_{2}$	[0, 1]
	Convergence factor α	[0, 2]
	Constant coefficient b	1
	Disordered solution l	[−1, 1]
SCWOA	Disordered solution $r_{1}$	[0, 1]
	Disordered solution $r_{2}$	[0, 1]
	Convergence factor α	[0, 2]
	Constant coefficient b	1
	Disordered solution l	[−1, 1]
	Constant a	2
	Disordered solution $r_{2}$	[0, 2π]
	Disordered solution $r_{3}$	[−2, 2]
	Disordered solution $r_{4}$	[0, 1]

**Table 3:** Simulation results of unimodal functions.

Function	Result	BA	CapSA	MFO	MVO	SAO	MDWA	WOA	SCWOA	Rank
$f_{1}$	Best	0.001159	5.36$\times 10^{-22}$	4.42$\times 10^{-6}$	0.092868	5.56$\times 10^{-5}$	9.20$\times 10^{-17}$	1.5$\times 10^{-192}$	0	1
	Worst	0.001622	8.63$\times 10^{-18}$	20000.00	0.359012	0.007819	2.41$\times 10^{-14}$	8.8$\times 10^{-170}$	0	
	Mean	0.001375	1.15$\times 10^{-18}$	2000.000	0.192690	0.001067	4.70$\times 10^{-15}$	3.3$\times 10^{-171}$	0	
	Std	0.000136	2.14$\times 10^{-18}$	4842.342	0.065528	0.001461	6.10$\times 10^{-15}$	0	0	
$f_{2}$	Best	0.143627	7.53$\times 10^{-12}$	5.34$\times 10^{-5}$	0.164170	0.040695	4.05$\times 10^{-9}$	4.9$\times 10^{-117}$	0	1
	Worst	1.486102	1.65$\times 10^{-9}$	60.00000	0.561635	0.322152	6.99$\times 10^{-8}$	6.8$\times 10^{-106}$	0	
	Mean	0.362503	3.36$\times 10^{-10}$	32.00004	0.297294	0.126300	1.80$\times 10^{-8}$	4.6$\times 10^{-107}$	0	
	Std	0.310119	4.01$\times 10^{-10}$	19.19046	0.080973	0.071809	1.46$\times 10^{-8}$	1.5$\times 10^{-106}$	0	
$f_{3}$	Best	0.002828	1.73$\times 10^{-19}$	361.1944	7.282789	0.019636	1.24$\times 10^{-7}$	1157.308	0	1
	Worst	0.007281	3.46$\times 10^{-15}$	43673.20	35.59056	68711.49	6.88$\times 10^{-5}$	26934.37	0	
	Mean	0.005109	3.75$\times 10^{-16}$	16485.94	16.95300	8723.780	1.03$\times 10^{-5}$	12424.99	0	
	Std	0.001217	7.74$\times 10^{-16}$	12964.40	7.354255	15170.22	1.94$\times 10^{-5}$	6176.585	0	
$f_{4}$	Best	0.014087	4.13$\times 10^{-12}$	26.51955	0.281197	0.002479	1.29$\times 10^{-6}$	0.001146	0	1
	Worst	0.027422	7.12$\times 10^{-10}$	74.51734	1.083297	0.021852	6.39$\times 10^{-5}$	83.74666	0	
	Mean	0.018084	1.65$\times 10^{-10}$	52.37642	0.613720	0.008145	1.31$\times 10^{-5}$	26.99115	0	
	Std	0.002698	1.73$\times 10^{-10}$	12.46703	0.222156	0.004628	1.34$\times 10^{-5}$	25.65131	0	
$f_{5}$	Best	22.68025	1.60$\times 10^{-9}$	24.41747	27.27617	0.001503	22.18621	25.89678	25.58608	4
	Worst	29.52813	9.42$\times 10^{-6}$	90079.05	2449.732	0.298228	88.62291	27.02118	28.57163	
	Mean	27.39530	9.80$\times 10^{-7}$	15426.22	408.6292	0.091653	33.16911	26.49432	27.21915	
	Std	1.590533	1.86$\times 10^{-6}$	33959.41	672.6372	0.079147	18.13722	0.308641	0.525503	
$f_{6}$	Best	0.013535	0.047475	0.018017	0.003399	0.001207	0.000658	8.93$\times 10^{-6}$	1.33$\times 10^{-7}$	1
	Worst	0.070121	0.955523	26.86892	0.032807	0.118872	0.011541	0.005396	5.06$\times 10^{-6}$	
	Mean	0.039194	0.529563	1.848647	0.014375	0.016951	0.004033	0.000751	1.50$\times 10^{-6}$	
	Std	0.013420	0.293325	5.042738	0.007323	0.021682	0.002478	0.001026	1.16$\times 10^{-6}$	

**Table 4:** Simulation results of multimodal functions.

Function	Result	BA	CapSA	MFO	MVO	SAO	MDWA	WOA	SCWOA	Rank
$f_{7}$	Best	18.13550	0	57.70755	59.76117	0.005055	0	0	0	1
	Worst	42.08115	0	219.1031	143.3769	208.2966	1.51$\times 10^{-11}$	0	0	
	Mean	29.38550	0	139.8432	104.8220	13.16234	1.77$\times 10^{-12}$	0	0	
	Std	6.161807	0	40.22221	24.85516	38.81178	3.03$\times 10^{-12}$	0	0	
$f_{8}$	Best	2.122723	1.41$\times 10^{-11}$	7.20$\times 10^{-4}$	0.103777	0.004603	6.24$\times 10^{-9}$	8.88$\times 10^{-16}$	8.88$\times 10^{-16}$	1
	Worst	3.225303	7.86$\times 10^{-10}$	19.96283	1.817965	0.593776	1.60$\times 10^{-7}$	7.99$\times 10^{-15}$	8.88$\times 10^{-16}$	
	Mean	2.683390	1.70$\times 10^{-10}$	10.62669	0.643666	0.037979	4.64$\times 10^{-8}$	4.32$\times 10^{-15}$	8.88$\times 10^{-16}$	
	Std	0.295728	1.82$\times 10^{-10}$	9.346467	0.585947	0.105724	3.68$\times 10^{-8}$	2.55$\times 10^{-15}$	0	
$f_{9}$	Best	5.18$\times 10^{-5}$	0	9.94$\times 10^{-6}$	0.205425	9.44$\times 10^{-6}$	0	0	0	1
	Worst	9.63$\times 10^{-5}$	0	90.51281	0.652374	12.51340	0.007407	0.073058	0	
	Mean	7.37$\times 10^{-5}$	0	18.05093	0.428925	1.112332	0.000247	0.005121	0	
	Std	1.14$\times 10^{-5}$	0	36.69684	0.112703	2.882955	0.001352	0.016458	0	
$f_{10}$	Best	8.75$\times 10^{-6}$	1.10$\times 10^{-13}$	1.87$\times 10^{-5}$	1.09$\times 10^{-3}$	1.06$\times 10^{-6}$	0.005529	0.000197	0.027374	5
	Worst	1.56$\times 10^{-5}$	4.60$\times 10^{-11}$	1.438733	4.361125	14.41869	0.015736	0.002311	0.145999	
	Mean	1.28$\times 10^{-5}$	8.43$\times 10^{-12}$	0.311838	1.166543	1.245729	0.010707	0.000483	0.073446	
	Std	1.98$\times 10^{-6}$	1.05$\times 10^{-11}$	0.422497	1.122964	2.863611	0.002669	0.000441	0.025835	

**Table 5:** Simulation results of fixed-dimension multimodal functions.

Function	Result	BA	CapSA	MFO	MVO	SAO	MDWA	WOA	SCWOA	Rank
$f_{11}$	Best	0.998004	0.998004	0.998004	0.998004	0.998004	0.998004	0.998004	0.998004	3
	Worst	12.67051	0.998004	5.928845	0.998004	11.72054	6.903342	10.76318	2.982105	
	Mean	10.19202	0.998004	1.394041	0.998004	3.579868	4.592604	1.588057	1.064298	
	Std	3.783086	1.49$\times 10^{-16}$	1.024618	5.84$\times 10^{-12}$	2.187821	2.453027	1.863075	0.362216	
$f_{12}$	Best	0.000308	0.000307	0.000457	0.000407	0.000410	0.000316	0.000309	0.000308	1
	Worst	0.001660	0.001223	0.002237	0.020363	0.014274	0.020364	0.002176	0.000330	
	Mean	0.000649	0.000430	0.000979	0.007278	0.002660	0.001933	0.000597	0.000315	
	Std	0.000499	0.000317	0.000416	0.009413	0.003165	0.005024	0.000409	5.24 × 10^−6^	
$f_{13}$	Best	−1	−1	−1	−1	−0.99988	−1	−1	−1	1
	Worst	−0.78575	−1	−0.93625	−1	−0.93625	−1	−0.93625	−1	
	Mean	−0.93046	−1	−0.97662	−1	−0.97136	−1	−0.98512	−1	
	Std	0.042517	0	0.031248	4.58 × 10^−7^	0.029805	0	0.027426	0	
$f_{14}$	Best	−10.5364	−10.5364	−10.5364	−10.5364	−10.5358	−10.5364	−10.5364	−10.5364	4
	Worst	−2.87114	−10.5364	−2.42173	−2.42733	−5.12804	−1.85948	−2.80656	−5.11863	
	Mean	−5.37063	−10.5364	−9.28154	−9.13569	−9.80391	−6.55031	−8.82645	−8.13141	
	Std	1.479732	2.56$\times 10^{-15}$	2.593989	2.898399	1.852026	3.203155	2.665397	2.521657	
$f_{15}$	Best	−1	−1	−1	−1	−0.99028	−1	−1	−1	1
	Worst	−0.99028	−1	−0.99028	−0.99028	−0.87301	−1	−0.99028	−1	
	Mean	−0.99644	−1	−0.99126	−0.99967	−0.97129	−1	−0.99644	−1	
	Std	0.004762	0	0.002965	0.001773	0.031843	0	0.004762	0	
$f_{16}$	Best	0.001669	2.03$\times 10^{-14}$	1.11$\times 10^{-15}$	0.005771	0.000755	6.83$\times 10^{-23}$	3.3$\times 10^{-124}$	0	1
	Worst	0.003717	1.01$\times 10^{-10}$	4.440211	0.345920	3.664003	6.88$\times 10^{-5}$	3.892485	0	
	Mean	0.002333	8.64$\times 10^{-12}$	0.148007	0.112455	0.126119	1.45$\times 10^{-5}$	0.489671	0	
	Std	0.000388	1.95$\times 10^{-11}$	0.810668	0.096192	0.668206	2.04$\times 10^{-5}$	1.117403	0	

**Table 6:** Results of the *p*-value Wilcoxon rank-sum.

Function	BA	CapSA	MFO	MVO	SAO	MDWA	WOA
$f_{1}$	1.21$\times 10^{-12}$	1.21$\times 10^{-12}$	1.21$\times 10^{-12}$	1.21$\times 10^{-12}$	1.21$\times 10^{-12}$	1.21$\times 10^{-12}$	1.21$\times 10^{-12}$
$f_{2}$	1.21$\times 10^{-12}$	1.21$\times 10^{-12}$	1.21$\times 10^{-12}$	1.21$\times 10^{-12}$	1.21$\times 10^{-12}$	1.21$\times 10^{-12}$	1.21$\times 10^{-12}$
$f_{3}$	1.21$\times 10^{-12}$	1.21$\times 10^{-12}$	1.21$\times 10^{-12}$	1.21$\times 10^{-12}$	1.21$\times 10^{-12}$	1.21$\times 10^{-12}$	1.21$\times 10^{-12}$
$f_{4}$	1.21$\times 10^{-12}$	1.21$\times 10^{-12}$	1.21$\times 10^{-12}$	1.21$\times 10^{-12}$	1.21$\times 10^{-12}$	1.21$\times 10^{-12}$	1.21$\times 10^{-12}$
$f_{5}$	N/A	3.02$\times 10^{-11}$	8.48$\times 10^{-9}$	1.61$\times 10^{-10}$	3.02$\times 10^{-11}$	1.84$\times 10^{-2}$	6.52$\times 10^{-9}$
$f_{6}$	3.02$\times 10^{-11}$	3.02$\times 10^{-11}$	3.02$\times 10^{-11}$	3.02$\times 10^{-11}$	3.02$\times 10^{-11}$	3.02$\times 10^{-11}$	3.02$\times 10^{-11}$
$f_{7}$	1.21$\times 10^{-12}$	N/A	1.21$\times 10^{-12}$	1.21$\times 10^{-12}$	1.21$\times 10^{-12}$	4.52$\times 10^{-12}$	N/A
$f_{8}$	1.21$\times 10^{-12}$	1.21$\times 10^{-12}$	1.21$\times 10^{-12}$	1.21$\times 10^{-12}$	1.21$\times 10^{-12}$	1.21$\times 10^{-12}$	1.16$\times 10^{-8}$
$f_{9}$	1.21$\times 10^{-12}$	N/A	1.21$\times 10^{-12}$	1.21$\times 10^{-12}$	1.21$\times 10^{-12}$	1.10$\times 10^{-2}$	8.15$\times 10^{-4}$
$f_{10}$	3.02$\times 10^{-11}$	3.02$\times 10^{-11}$	N/A	1.31$\times 10^{-8}$	1.95$\times 10^{-3}$	3.02$\times 10^{-11}$	3.02$\times 10^{-11}$
$f_{11}$	6.12$\times 10^{-10}$	1.41$\times 10^{-11}$	2.73$\times 10^{-5}$	3.02$\times 10^{-11}$	1.87$\times 10^{-7}$	7.69$\times 10^{-8}$	8.29$\times 10^{-6}$
$f_{12}$	8.53$\times 10^{-4}$	1.09$\times 10^{-6}$	3.02$\times 10^{-11}$	3.02$\times 10^{-11}$	3.02$\times 10^{-11}$	2.15$\times 10^{-10}$	5.09$\times 10^{-8}$
$f_{13}$	1.21$\times 10^{-12}$	N/A	3.06$\times 10^{-4}$	1.21$\times 10^{-12}$	1.21$\times 10^{-12}$	N/A	5.54$\times 10^{-3}$
$f_{14}$	2.52$\times 10^{-2}$	1.36$\times 10^{-11}$	4.98$\times 10^{-6}$	1.43$\times 10^{-5}$	6.01$\times 10^{-8}$	8.77$\times 10^{-1}$	6.77$\times 10^{-5}$
$f_{15}$	1.21$\times 10^{-12}$	N/A	3.94$\times 10^{-12}$	1.21$\times 10^{-12}$	1.21$\times 10^{-12}$	N/A	3.08$\times 10^{-4}$
$f_{16}$	1.21$\times 10^{-12}$	1.21$\times 10^{-12}$	1.21$\times 10^{-12}$	1.21$\times 10^{-12}$	1.21$\times 10^{-12}$	1.21$\times 10^{-12}$	1.21$\times 10^{-12}$

**Table 7:** Comparison results of the three-bar truss design.

Algorithm	Optimal Value for Elements		Optimal Cost
	*A*_1_	*A*_2_	
GWO [39]	0.788648	0.408325	263.8960063
CS [40]	0.78867	0.40902	263.9716
MFO [3]	0.78824477	0.4094669	263.8959796
Ray and Sain [41]	0.795	0.395	264.3
AOA [42]	0.79369	0.39426	263.9154
Raj et al. [43]	0.789764410	0.405176050	263.89671
Das et al. [44]	0.78867	0.40902	263.9716
GEO [45]	0.79369	0.39426	263.9154
RFO [46]	0.75356	0.55373	268.51195
GSA [34]	0.747070495056356	0.530675746732991	264.769804538555
ESOA [47]	0.788192	0.409618	263.896
DE [47]	0.788675	0.408248	263.896
L-Shade [47]	0.78867514	0.40824829	263.896
MPEDE [47]	0.78924889	0.40662803	263.896
HGSO [48]	0.778254	0.440528	264.1762
HGS [48]	0.7884562	0.40886831	263.8959
SC-GWO [49]	0.78941	0.40617	263.8963
COA [31]	0.788057	0.410073	263.903379
MRA [50]	0.788574	0.408536	263.8959
AO-TSA [51]	0.790512	0.403105	263.9010
TSA [51]	0.797520	0.387339	264.3067
I-GWO [51]	0.784408	0.420579	263.9220
BO [51]	0.792187	0.398517	263.9159
KH [35]	0.785125499417041	0.420705357829172	264.137561671
BOA [35]	0.823331535298134	0.313381441824923	266.734135381
SELO [52]	0.7878	0.4108	263.8964
HBO [52]	0.7887	0.4082	263.8959
LFD [52]	0.7879	0.4106	263.8963
KABC [53]	0.7886	0.4084	263.8959
SCWOA	0.788674	0.408234	263.895843

**Table 8:** Comparison results of the tubular column design.

Algorithm	Optimal Value for Elements		Optimal Cost
	*d*	*t*	
CS [54]	5.45139	0.29196	26.53217
ISA [55]	5.45115623	0.29196547	26.5313
SNS [56]	5.45115632	0.29196547	26.4994969
Rao [57]	5.44	0.293	26.5323
Gandomi [40]	5.45139	0.29196	26.5321
CSA [58]	5.451163397	0.291965509	26.531364472
MFPA [59]	5.4512	0.29197	26.49995
GSA-GA [60]	5.45115623	0.29196548	26.531328
AGQPSO [61]	5.451156	0.29196	26.531328
FPA [62]	5.45116	0.291965	26.49948
KH [32]	5.451278	0.291957	26.5314
BOA [32]	5.448426	0.292463	26.512782
HFBOA [32]	5.451157	0.291966	26.499503
Rocha and Fernandes [63]	5.45139	0.29199	26.53227
EM [64]	5.452383	0.29190	26.53380
HEM [64]	5.451083	0.29199	26.53227
KOA [31]	5.4512	0.2920	26.499497
FLA [31]	5.4801	0.2905	26.563266
COA [31]	5.4511	0.2920	26.501823
GTO [31]	5.4512	0.2920	26.499497
RUN [31]	5.4512	0.2920	26.499497
GWO [31]	5.4511	0.2920	26.499770
SMA [31]	5.4512	0.2920	26.499538
DO [31]	5.4512	0.2920	26.499497
POA [31]	5.4512	0.2920	26.499497
FA [65]	N/A	N/A	26.5200
AOS [65]	N/A	N/A	26.5313783
SCWOA	5.5537	0.2502	25.5346

**Table 9:** Comparison results of the speed reducer design.

Algorithm	Optimal Values for Elements							Optimal Cost
	b	m	z	$l_{1}$	$l_{2}$	$d_{1}$	$d_{2}$	
APSO [66]	3.50131	0.7	18	8.12781	8.04212	3.35245	5.28708	3187.63049
GA [67]	3.510253	0.7	17	8.35	7.8	3.362201	5.287723	3067.561
SES [68]	3.506163	0.700831	17	7.460181	7.962143	3.3629	5.308949	3025.005127
PSO [69]	3.5001	0.7	17	7.5177	7.7832	3.3508	5.2867	3145.922
GSA [70]	3.6	0.7	17	8.3	7.8	3.369658	5.289224	3051.12
hHHO-SCA [71]	3.506119	0.7	17	7.3	7.99141	3.452569	5.286749	3029.873076
MDA [72]	3.5	0.7	17	7.3	7.670396	3.542421	5.245814	3019.583365
SCA [29]	3.508755	0.7	17	7.3	7.8	3.46102	5.289213	3030.563
HS [73]	3.520124	0.7	17	8.37	7.8	3.36697	5.288719	3029.002
HIS [74]	3.520124	0.7	17	8.37	7.8	3.36697	5.288719	3029.002
GSA [75]	3.6	0.7	17	8.3	7.802442	3.369658	5.289224	3051.1209
EA [68]	3.506163	0.700831	17	7.46018	7.962143	3.3629	5.3090	3025.005
CMA-ES [76]	2.6	0.8	17	7.3	7.8	2.9	5	8962.48
L-SHADE [76]	3.4367	0.7179	17.2544	8.1541	7.9808	3.2999	5.3498	7361.25
EHO [76]	3.4889	0.7782	23.2193	7.849	8.1021	3.5603	5.2459	73504.7
GOA [76]	3.5126	0.7033	17.2246	7.9131	7.9627	3.6567	5.2784	3169.32
TEO [76]	3.4261	0.7	17.6222	7.7408	7.9775	3.4145	5.2758	3595.59
TLBO [77]	3.508755	0.7	17	7.3	7.8	3.46102	5.2892113	3030.563
BWO [34]	3.58	0.72	18.28	7.73	7.73	3.43	5.28	3417.1535
DE [78]	3.520124	0.7	17	8.37	7.8	3.36697	5.288719	3029.002
INFO [79]	3.514301	0.7	17	7.307301	7.8078	3.466456	5.29752	3036.931
CPA [79]	3.525688	0.7	17	8.378957	7.8078	3.372258	5.29702	3035.367
BOA [80]	3.5239	0.7003	17.0088	8.0962	8.004	3.4048	5.3286	3061.6
HIWOA [80]	3.5605	0.7	17	7.3	8.1169	3.4631	5.2913	3059.6
PSCA [81]	3.54562	0.7	17.0023	8.3	8.3	3.37846	5.27946	3038.885
HOA [82]	3.56008	0.7	17	7.34912	7.8	3.49325	5.28415	3058.577
ES [83]	3.506163	0.700831	17	7.460181	7.962143	3.3629	5.309	3025.005
CKGSA [33]	3.5926	0.7134	17.1221	7.7464	8.1030	3.4464	5.3013	3163.2207
SCWOA	3.50228	0.7	17	7.88793	7.82363	3.36347	5.29537	3017.596

**Table 11:** Comparison results of the tension/compression spring design.

Algorithm	Optimal Value for Elements			Optimal Cost
	d	D	N	
SFOA [87]	0.051800	0.359000	11.279000	0.012700
APSO [66]	0.052588	0.378343	10.138862	0.012700
GSA [88]	0.050276	0.323680	13.525410	0.0127022
CC [89]	0.050000	0.315900	14.250000	0.0128334
GA [90]	0.051480	0.351661	11.632201	0.01270478
MVO [4]	0.05251	0.37602	10.33513	0.012790
Arora [91]	0.053396	0.399180	9.185400	0.012730
SA [76]	0.0570	0.4953	6.2225	0.01321
CMA-ES [76]	0.0973	1.1488	13.54530	0.85621
GOA [76]	0.0516	0.3360	13.500	0.01389
HHO [76]	0.0570	0.4991	6.2180	0.01281
TLBO [77]	0.050780	0.334779	12.72269	0.012709667
CSO [34]	0.0671	0.8482	2.4074	0.01682958
SCSO [34]	0.0500	0.3175	14.0200	0.012717020
SCA [92]	0.050780	0.334779	12.72269	0.012709667
hHHO-SCA [93]	0.054693	0.433378	7.891402	0.0128229
RFO [46]	0.05189	0.36142	11.58436	0.01321
LSA [94]	0.05027598	0.3236795	13.52541	0.01272045
CA [95]	0.05	0.317395	14.031795	0.012721
SI [96]	0.050417	0.321532	13.97991	0.01306
ESOA [47]	0.05	0.317168	14.0715	0.01274345
MPEDE [47]	0.05956062	0.5767404	4.71717282	0.01374
HGS [48]	0.05	0.3174	14.0306	0.0127
FLA [48]	0.0499	0.315	14.3045	0.0127
COA [31]	0.05	0.31137	14.862261	0.0131260069
RUN [31]	0.053107	0.391807	9.493688	0.0127011107
I-GWO [51]	0.050773	0.334713	12.77824	0.012803
FA [97]	0.052459	0.356839	11.130281	0.012894
CRCC [97]	0.05	0.3159	14.25	0.012833
PF [97]	0.053396	0.39918	9.1854	0.01273
PSCA [81]	0.05	0.317407	14.1166	0.012789
CASFO [36]	0.1413	1.3627	10.9889	3.6387
SFO [36]	0.1406	1.3608	10.92481	3.6477
CLPSO [35]	0.0528162	0.38365734	9.9234572	0.01276085
VPPSO [98]	0.0525	0.3756	10.2659	0.0127
KABC [53]	0.0556	0.4575	7.148	0.013017
SCWOA	0.054627	0.325243	11.654662	0.0126653

**Table 10:** Comparison results of the piston lever design.

Algorithm	Optimal Value for Elements				Optimal Cost
	H	B	X	D	
PSO [84]	133.3	2.44	117.14	4.75	122
DE [84]	129.4	2.43	119.8	4.75	159
GA [84]	250	3.96	60.03	5.91	161
HPSO [84]	135.5	2.48	116.62	4.75	162
CS [54]	0.050	2.043	120	4.085	8.427
SNS [56]	0.050	2.042	120	4.083	8.412698349
SCSO [34]	0.050	2.040	119.99	4.083	8.40901438899551
CSO [34]	0.050	2.399	85.68	4.0804	13.7094866557362
GWO [34]	0.060	2.0390	120	4.083	8.40908765909047
WAO [34]	0.099	2.057	118.4	4.112	9.05943208079399
SSA [34]	0.050	2.073	116.32	4.145	8.80243253777633
GSA [34]	497.49	500	60.041	2.215	168.094363238712
BWO [34]	12.364	12.801	172.02	3.074	95.9980864948937
AOS [85]	0.05	2.042112482	119.951727	4.084004492	8.419142742
GTO [86]	0.05	2.052859	119.6392	4.089713	8.41270
MFO [86]	0.05	2.041514	120	4.083365	8.412698
WOA [86]	0.051874	2.045915	119.9579	4.085849	8.449975
DMOA [65]	0.05	0.125073578	120	4.116042166	4.695
AOA [65]	0.05	0.125073578	120	4.116042166	7.738
CPSOGSA [65]	500	500	120	2.578147082	4.6949
BBO [65]	129.4	2.43	119.8	4.75	4.6956
ISA [65]	N/A	N/A	N/A	N/A	8.4
CGO [65]	N/A	N/A	N/A	N/A	8.41281381
MGA [65]	N/A	N/A	N/A	N/A	8.41340665
SCWOA	0.05	0.138542	120	4.116025	4.6827

**Table 12:** Comparison results of the welded beam design.

Algorithm	Optimal Value for Elements				Optimal Cost
	*h*	*l*	*t*	*b*	
BBO [39]	0.1854860	4.3129000	8.4399030	0.2359020	1.9180550
PSO [39]	0.219292	3.430416	8.433559	0.236204	1.852720
GSA [88]	0.182129	3.856979	10.000	0.202376	1.87995
RO [99]	0.203687	3.528467	9.004233	0.207241	1.735344
CSCA [100]	0.203137	3.542998	9.033498	0.206179	1.733461
GA [101]	0.2489	6.1730	8.1789	0.2533	2.4300
DAVID [102]	0.2434	6.2552	8.2915	0.2444	2.3841
SIMPLEX [102]	0.2792	5.6256	7.7512	0.2796	2.5307
APPROX [102]	0.2444	6.2189	8.2915	0.2444	2.3815
HS [103]	0.2442	6.2231	8.2915	0.2400	2.3807
SCA [29]	0.204695	3.536291	9.004290	0.210025	1.759173
ES [104]	0.199742	3.612060	9.037500	0.20682	1.73730
CS [40]	0.2015	3.562	9.0414	0.2057	1.73121
Coello [105]	0.208800	3.420500	8.997500	0.2100	1.74831
CMA-ES [76]	0.5617	4.3786	4.6772	0.9286	2.28384
L-SHADE [76]	0.4819	3.2140	5.4763	0.5753	3.43372
EHO [76]	1.0149	4.7616	4.8130	0.8722	3.36770
GOA [76]	0.4069	2.1411	6.3834	0.4123	2.43534
HHO [76]	0.1961	3.7449	9.0061	0.2071	1.75163
TLBO [77]	0.204695	3.536291	9.004290	0.210025	1.759173
CSO [34]	0.2044	3.3125	8.9941	0.2108	1.7321
Random [102]	0.4575	4.7313	5.0853	0.6600	4.11856
Ragsdell [102]	0.2455	6.1960	8.2730	0.2455	2.38594
Siddall [106]	0.2444	6.2189	8.2915	0.2444	2.38154
DDSCA [107]	0.20516	3.4759	9.0797	0.20552	1.7305
hHHO-SCA [71]	0.190086	3.696496	9.386343	0.204157	1.779032
WWO [108]	0.22214	3.67812	8.84965	0.23489	1.96842
NMDE [109]	0.2450054	6.284511	8.19911	2.450054	2.377135
SaDE [110]	0.306	3.02	6.33	0.419	2.48
PSOGSA [110]	0.24	3.09	8.36	0.24	1.99
HGSA [110]	0.211	3.40	8.90	0.212	1.75
ACVO [110]	0.205	3.48	9.04	0.206	1.73
HPSO [95]	0.20573	3.470489	9.036624	0.20573	1.728024
CDE [95]	0.203137	3.542998	9.033498	0.206179	1.733462
SBM [96]	0.2407	6.4851	8.2399	0.2497	2.4426
BFOA [96]	0.2057	3.4711	9.0367	0.2057	2.3868
EA [96]	0.2443	6.2201	8.2940	0.2444	2.3816
T-Cell [96]	0.2444	6.1286	8.2915	0.2444	2.3811
FSA [96]	0.2444	6.1258	8.2939	0.2444	2.3811
IPSO [96]	0.2444	6.2175	8.2915	0.2444	2.3810
DSS-DE [96]	0.2444	6.1275	8.2915	0.2444	2.3810
HSA-GA [96]	0.2231	1.5815	12.8468	0.2445	2.2500
FLA [48]	0.1983	3.6664	9.0705	0.2057	1.75
HGS [111]	0.26	5.1025	8.03961	0.26	2.302076
LFD [49]	0.1857	3.9070	9.1552	0.2051	1.7700
COA [31]	0.174041	7.087014	8.997138	0.207648	2.1324620263
LA [112]	0.2213	3.2818	8.7579	0.2216	1.8446
FA [6]	0.201762	6.804895	9.627042	0.205249	2.2837
MDWA [6]	0.203494	7.244195	9.058998	0.206944	2.2474
EBSCA [113]	0.17758	4.7517	9.0406	0.20573	1.8435
SFO [36]	0.2038	3.6630	9.0506	0.2064	1.73231
EEGWO [22]	0.2444	0.2444	8.2928	0.2444	2.3813
RCGA [22]	N/A	N/A	N/A	N/A	2.381133
QHGSO [114]	0.2152	6.8889	8.815	0.216	2.2864
MCSS [114]	0.2434	6.2552	8.2915	0.2444	2.3841
BA [115]	2	0.1	3.174303	2	1.818138
CLPSO [35]	0.20043684951	3.61781217135	9.12632634245	0.205392564	1.74936011824
SCWOA	0.205657	3.251177	9.039105	0.205468	1.69682

**Table 13:** Comparison results of the gear train design.

Algorithm	Optimal Value for Elements				Optimal Cost
	$n_{A}$	$n_{B}$	$n_{C}$	$n_{D}$	
GA [39]	49	19	16	43	2.70$\times 10^{-12}$
PSO [39]	34	13	20	53	2.31$\times 10^{-11}$
ICA [39]	43	16	19	49	2.70$\times 10^{-12}$
BBO [39]	53	26	15	51	2.31$\times 10^{-11}$
NNA [39]	49	16	19	43	2.70$\times 10^{-12}$
GWO [39]	49	19	16	43	2.70$\times 10^{-12}$
WSA [39]	43	16	19	49	2.70$\times 10^{-12}$
CS [54]	43	16	19	49	2.70$\times 10^{-12}$
ABC [116]	49	16	19	43	2.70$\times 10^{-12}$
MSFWA [117]	49	19	16	43	2.70$\times 10^{-12}$
MBA [118]	43	16	19	49	2.70$\times 10^{-12}$
ISA [55]	43	19	16	49	2.70$\times 10^{-12}$
APSO [66]	43	16	19	49	2.70$\times 10^{-12}$
IAPSO [66]	43	16	19	49	2.70$\times 10^{-12}$
MVO [4]	43	16	19	49	2.70$\times 10^{-12}$
MFO [3]	43	19	16	49	2.70$\times 10^{-12}$
ALO [119]	49	19	16	43	2.70$\times 10^{-12}$
PSOSCALF [120]	49	19	16	43	2.70$\times 10^{-12}$
SNS [56]	43	19	16	49	2.70085714$\times 10^{-12}$
Sandgren [121]	45	22	18	60	5.712$\times 10^{-6}$
Kannan and Kramer [122]	33	15	13	41	2.146$\times 10^{-8}$
Deb and Goya [123]	49	16	19	43	2.701$\times 10^{-12}$
Gandomi er al. [40]	43	16	19	49	2.701$\times 10^{-12}$
CSA [58]	43	16	19	49	2.701$\times 10^{-12}$
ALM [122]	33	15	13	41	2.1469$\times 10^{-8}$
MFPA [59]	60	28	17	55	3.69$\times 10^{-5}$
FDA [124]	49	19	16	43	2.7008571$\times 10^{-12}$
CAPSO [125]	49	19	16	43	2.701$\times 10^{-12}$
GeneAS [125]	33	14	17	50	1.362$\times 10^{-9}$
BOA [125]	43	16	19	49	2.701$\times 10^{-12}$
Simulated annealing [125]	52	15	30	60	2.36$\times 10^{-9}$
Sequential linearization approach [125]	42	16	19	50	2.3$\times 10^{-7}$
Mixed-variable evolutionary programming [125]	52	15	30	60	2.36$\times 10^{-9}$
Mixed integer discrete continuous programming [125]	47	29	14	59	4.5$\times 10^{-6}$
Mixed integer discrete continuous optimization [125]	33	15	13	41	2.146$\times 10^{-8}$
Nonlinear integer and discrete programming [125]	45	22	18	60	5.712$\times 10^{-6}$
BO [126]	43	19	16	49	2.700857$\times 10^{-12}$
KOA [31]	44	20	16	50	2.700857$\times 10^{-12}$
FLA [31]	44	16	20	49	2.700857$\times 10^{-12}$
COA [31]	23	14	12	48	9.92158$\times 10^{-10}$
RUN [31]	44	17	19	49	2.700857$\times 10^{-12}$
SMA [31]	52	30	13	53	2.307816$\times 10^{-11}$
DO [31]	49	16	19	44	2.700857$\times 10^{-12}$
POA [31]	44	17	19	49	2.70085$\times 10^{-12}$
PDO [65]	48	17	22	54	2.70$\times 10^{-12}$
DMOA [65]	49	19	16	43	2.70$\times 10^{-12}$
AOA [65]	49	19	19	54	2.70$\times 10^{-12}$
CPSOGSA [65]	55	16	16	43	2.31$\times 10^{-11}$
SSA [65]	49	19	19	49	2.70$\times 10^{-12}$
SCA [65]	49	19	34	49	2.700857$\times 10^{-12}$
IEHO [127]	19	16	43	49	2.70085$\times 10^{-12}$
MEWOA [37]	49	16	19	43	2.7099$\times 10^{-12}$
ARO [128]	49	19	16	43	2.7009$\times 10^{-12}$
BCA [129]	43	16	19	49	2.7009$\times 10^{-12}$
BWO [130]	50	18	17	46	7.5421$\times 10^{-17}$
GMO [53]	43	19	16	49	2.700857$\times 10^{-12}$
SCWOA	51	33	17	53	2.6574$\times 10^{-18}$

**Table 14:** Comparison results of the car side impact design.

Algorithm	Optimal Value for Elements						Optimal Cost
	$x_{1}$	$x_{2}$	$x_{3}$	$x_{4}$	$x_{5}$	$x_{6}$	
	$x_{7}$	$x_{8}$	$x_{9}$	$x_{10}$	$x_{11}$		
PSO [131]	0.5	1.1167	0.5	1.30208	0.5	1.5	
	0.5	0.345	0.192	−19.54935	−0.00431		22.84474
GA [131]	0.5	1.28017	0.50001	1.03302	0.50001	0.5	
	0.5	0.34994	0.192	10.3119	0.00167		22.85653
CS [54]	0.5	1.11643	0.5	1.30208	0.5	1.5	
	0.5	0.345	0.192	−19.54935	−0.00431		22.84294
BA [55]	0.5	1.1167	0.5	1.30208	0.5	1.5	
	0.5	0.345	0.192	−19.54935	−0.00431		22.84474
SNS [56]	0.5	1.115933208	0.5	1.302918991	0.5	1.5	
	0.5	0.345	0.192	−19.6388662	1.49192 × 10^−6^		22.84297965
DE [38]	0.5	1.1167	0.5	1.30208	0.5	1.5	
	0.5	0.345	0.192	−19.54935	−0.00431		22.84474
FA [38]	0.5	1.36	0.5	1.202	0.5	1.12	
	0.5	0.345	0.192	8.87307	−18.99808		22.84298
TLBO [38]	0.5	1.1135	0.5	1.307	0.5	1.5	
	0.5	0.345	0.192	−20.0655	0.1139		22.8436
TLCS [38]	0.5	1.1163	0.5	1.3023	0.5	1.5	
	0.5	0.345	0.192	−19.5721	0.0157		22.8430
CPA [38]	0.5	1.1157586	0.5	1.30321196	0.5	1.5	
	0.5	0.345	0.27247957	−19.67009727	0.00000206		22.84298982
ABC [132]	0.5	1.0624205	0.5148211	1.4491503	0.5	1.5	
	0.5	0.345	0.192	−29.34755	0.7410998		23.17588963
MFO [132]	0.5	1.116539	0.5	1.301908	0.5	1.5	
	0.5	0.345	0.345	−19.5304	−0.000006		22.84297087
ALO [132]	0.5	1.11596	0.5	1.30286	0.5	1.5	
	0.5	0.345	0.192	−19.6330	0.023649		22.84298071
ER-WCA [132]	0.5	1.118688	0.5	1.298407	0.5	1.5	
	0.5	0.345	0.192	−19.1461	−0.01527		22.84326462
GWO [132]	0.5	1.111484	0.5	1.312203	0.501214	1.5	
	0.5	0.345	0.192	−20.6057	−25531		22.85279276
WCA [132]	0.5	1.1155932	0.5	1.3034919	0.5000146	1.5	
	0.5	0.345	0.192	−19.69967	−0.023854		22.84303648
MBA [132]	0.5	1.1172701	0.5	1.30008438	0.5	1.499987	
	0.5	0.345	0.345	−19.40045	−0.379205		22.84359640
SSA [132]	0.5	1.1093195	0.5	1.3148	0.5	1.499999	
	0.5	0.345	0.192	−20.821793	0.4412962		22.84651410
WOA [132]	0.5	1.108001	0.534477	1.30577	0.5	1.473844	
	0.5	0.345	0.192	−19.69924	3.4816923		23.04216220
CSS [132]	0.5	1.184389	0.5	1.230036	0.5	1.5	
	0.5	0.280792	0.342425	−7.394733	0.042206		23.00733588
FACSS [133]	0.5	1.127288	0.5	1.285546	0.5	1.499999	
	0.5	0.344991	0.202079	−17.607749	8.297 × 10^−5^		22.84907401
GOA [134]	0.5	1.1167	0.5	1.30208	0.5	1.5	
	0.5	0.345	0.192	−19.54935	−0.00431		22.84474
HGOANM [134]	0.5	1.11643	0.5	1.30208	0.5	1.5	
	0.5	0.345	0.192	−19.54935	−0.00431		22.84294
EOBL-GOA [135]	0.5	1.11643	0.5	1.30208	0.5	1.5	
	0.5	0.345	0.192	−19.54935	−0.00431		22.84294
CLPSO [35]	0.5061	1.17379	0.5013	1.24706	0.5037	1.4956	
	0.5	0.345	0.345	−9.5985	3.3627		23.06244
ACO [35]	0.5	1.12004	0.5	1.29627	0.5	1.5	
	0.5	0.345	0.192	−18.905	−0.0008		22.84371
KH [35]	0.5	1.14747	0.5	1.26118	0.5	1.5	
	0.5	0.345	0.345	−13.998	−0.8984		22.88596
HHO [35]	0.5	1.15627	0.5	1.27133	0.5	1.4777	
	0.5	0.345	0.192	−14.592	−2.4898		22.98537
BOA [35]	0.8246	1.03224	0.54007	1.35639	0.6377	1.26889	
	0.5854	0.192	0.345	−5.7333	0.4352		25.06573
HGSO [35]	0.5	1.22375	0.5	1.27111	0.5	1.31085	
	0.5	0.345	0.345	−4.3235	2.93676		23.43457
LIACO [35]	0.5	1.11593	0.5	1.30293	0.5	1.5	
	0.5	0.192	0.345	−19.64	−0.000003		22.84299
SMO [35]	0.5	1.11634	0.5	1.30224	0.5	1.5	
	0.5	0.345	0.345	−19.566	0.000001		22.84298
SCWOA	0.5	1.11643	0.5	1.30178	0.5	1.5	
	0.5	0.345	0.192	−19.48754	−0.00453		22.84278

## Data Availability

The data presented in this study are available on request from the corresponding author.
